# Rational design of innate defense regulator peptides as tumor vaccine adjuvants

**DOI:** 10.1038/s41541-021-00334-3

**Published:** 2021-05-20

**Authors:** Yaomei Tian, Qiuyue Hu, Rui Zhang, Bailing Zhou, Daoyuan Xie, Yuanda Wang, Xueyan Zhang, Li Yang

**Affiliations:** 1 Department of Biotherapy, Cancer Center, West China Hospital, Sichuan University, Chengdu, Sichuan PR China; 2 College of Bioengineering, Sichuan University of Science & Engineering, Zigong, Sichuan PR China

**Keywords:** Cancer, Drug discovery, Immunology

## Abstract

The development of adjuvants has been an empirical process. Efforts to develop a new design and evaluation system for novel adjuvants are not only desirable but also necessary. Moreover, composite adjuvants that contain two or more types of adjuvants to synergistically enhance the immune response are important for adjuvant and vaccine design. Innate defense regulator peptides (IDRs) are promising adjuvants for clinical immunotherapy because they exhibit multifaceted immunomodulatory capabilities. However, the rational design and discovery of IDRs that have improved immunomodulatory activities have been hampered by the lack of screening techniques and the great challenges in the identification of their interaction partners. Here, we describe a screening and evaluation system for IDR design. On the basis of in vitro screening, the optimized IDR DP7 recruited neutrophils, monocytes and macrophages to the site of infection. The adjuvant, comprising the DP7 and CpG oligonucleotide (CpG), induced chemokine/cytokine expression, enhanced the antigen uptake by dendritic cells and upregulated surface marker expression in dendritic cells. Vaccination with the NY-ESO-1 or OVA antigens combined with the adjuvant alum/CpG/DP7 strongly suppressed tumor growth in mice which was due to the improvement of antigen-specific humoral and cellular immunity. Regarding the mechanism of action, GPR35 may be the potential interaction partner of DP7. Our study revealed the potential application of the screening and evaluation system as a strategy for rationally designing effective IDRs or composite adjuvants and identifying their mechanism of action.

## Introduction

The use of peptide/protein cancer vaccines has emerged as a valid active immunotherapeutic approach for clinical cancer treatment that mediates the tumor-associated antigen-specific immune response^1^. When peptide or protein antigens have inadequate immunogenicity, adjuvants are utilized that act as essential components in peptide/protein cancer vaccines to strengthen antigen immunogenicity and elicit an effective immune response^2^. However, only a few conventional adjuvants based on aluminum salts and oil-in-water emulsions have been licensed for use in human cancer vaccines^3^. Recently, CpG, as a vaccine adjuvant, has also been approved for use in a hepatitis B vaccine^4^. The development of conventional adjuvants mainly depends on empirical studies, and their mechanism of action is little known or only partially understood^5^. Recently, an increased understanding of the role of adjuvants in the innate immune response has resulted in the rapid development of new-generation adjuvant candidates^6^.

A single immune agonist is not always effective at eliciting an efficacious immune response^7^. For instance, aluminum hydroxide-based adjuvant vaccines tend to prime the Th2 immune response rather than a Th1-biased response. Composite adjuvates synergistically enhance the immune response^8,9^. According to the specific immune response generated by different types of adjuvants, several composite adjuvant systems show promise in clinical applications, such as AS01, AS04, and AS15^7^. It was found in our previous study that complexing immunomodulatory defense peptides with aluminum hydrogel/polysaccharides can enhance immune responses and antitumor effects^10^. Therefore, developing a new adjuvant or composite adjuvant and understanding the mechanism of action are crucial to vaccine efficacy.

HDPs, a class of natural peptides produced by all complex species, have attracted researchers’ attention due to their direct and broad antimicrobial activity^11^. More recently, the immunomodulatory effects of HDPs have been increasingly appreciated, including the induction of cytokine and chemokine expression and the promotion of wound healing, leukocyte activation, and macrophage/dendritic cell differentiation^12,13^. Consistent with their immunomodulatory properties, a variety of HDPs exhibit adjuvant-like activity by stimulating innate immune responses and modulating adaptive immunity^14^. However, the induction of mast cell degranulation and apoptosis and the high manufacturing cost have moderately hindered the development of HDPs^15^.

Innate defense regulator (IDR) peptides, which are synthetic and short peptide analogs of HDPs, exhibit powerful immunomodulatory activity. One of the first derivatives that used HDP as a template, IDR-1, could mediate the innate immune response to protect mice against bacterial infection^15^. Three other synthetic immunomodulatory peptides, IDR-HH2^10,16,17^, IDR-1002^18–20^, and IDR-1018^21^, exhibited prominent adjuvant activities due to their excellent immunomodulatory properties. Despite the strong adjuvant activities of IDRs and the abundant templates provided by HDPs, a screening and evaluation system for rationally designing and screening IDR adjuvants is lacking and in need of development. In our previous study, ten synthetic and short peptide analogs (named DP2-11) of Bac2A were identified via an amino acid-based activity prediction method, and their antimicrobial activities were compared in vivo and in vitro^22^. In further studies, we found that DP2-11 displayed varying degrees of immunomodulatory activity. Therefore, to identify the best defense peptide, a screening and evaluation system was built, in which DP7 exhibited improved immunomodulatory activity compared to the other analogs and acted as an effective vaccine adjuvant. However, the mechanism of action of DP7 is unknown.

The discoveries of previous studies regarding the mode of action of HDPs and IDRs have revealed the complexity of the underlying mechanisms and the diversity of the involved receptors^23^. LL-37 is the best-studied host defense peptide involved in the mechanism of action, and its cell membrane receptors include Gi protein-coupled receptors such as mas-related gene X2 (MRGX2)^24^ and formyl peptide receptor 2 (FPR2)^25^, type I insulin-like growth factor receptor (IGF-1R)^26^, purinergic receptor (P2X7)^27^, chemokine receptor (CXCR2)^28^ and epidermal growth factor receptor EGFR^29^. Recently, glyceraldehyde 3-phosphate dehydrogenase (GAPDH) was reported as an intracellular receptor of LL-37^30^. Based on the above studies, we speculate that DP7 may share surface receptors and intracellular receptors with LL-37. In this study, we used transcriptional sequencing followed by gene silencing to identify the Gi protein-coupled receptor GPR35 as a potential surface receptor for DP7.

Therefore, a complete screening and evaluation system consisting of a bioinformatic approach, in vitro screening, in vivo testing, and the determination of the mechanism of action was built. Based on the screening and evaluation system, we rationally screened the IDR with the best performance, DP7. DP7, which activates GPR35 in dendritic cells, synergizes with CpG to induce chemokine/cytokine expression, enhance the cellular uptake of antigen by dendritic cells and upregulate surface marker expression in dendritic cells. Vaccination with the NY-ESO-1 or OVA antigens and the composite adjuvant alum/CpG/DP7 strongly suppressed tumor growth in multiple tumor models.

## Results

### The IDR DP7 as the lead candidate

**Fig. 1:**
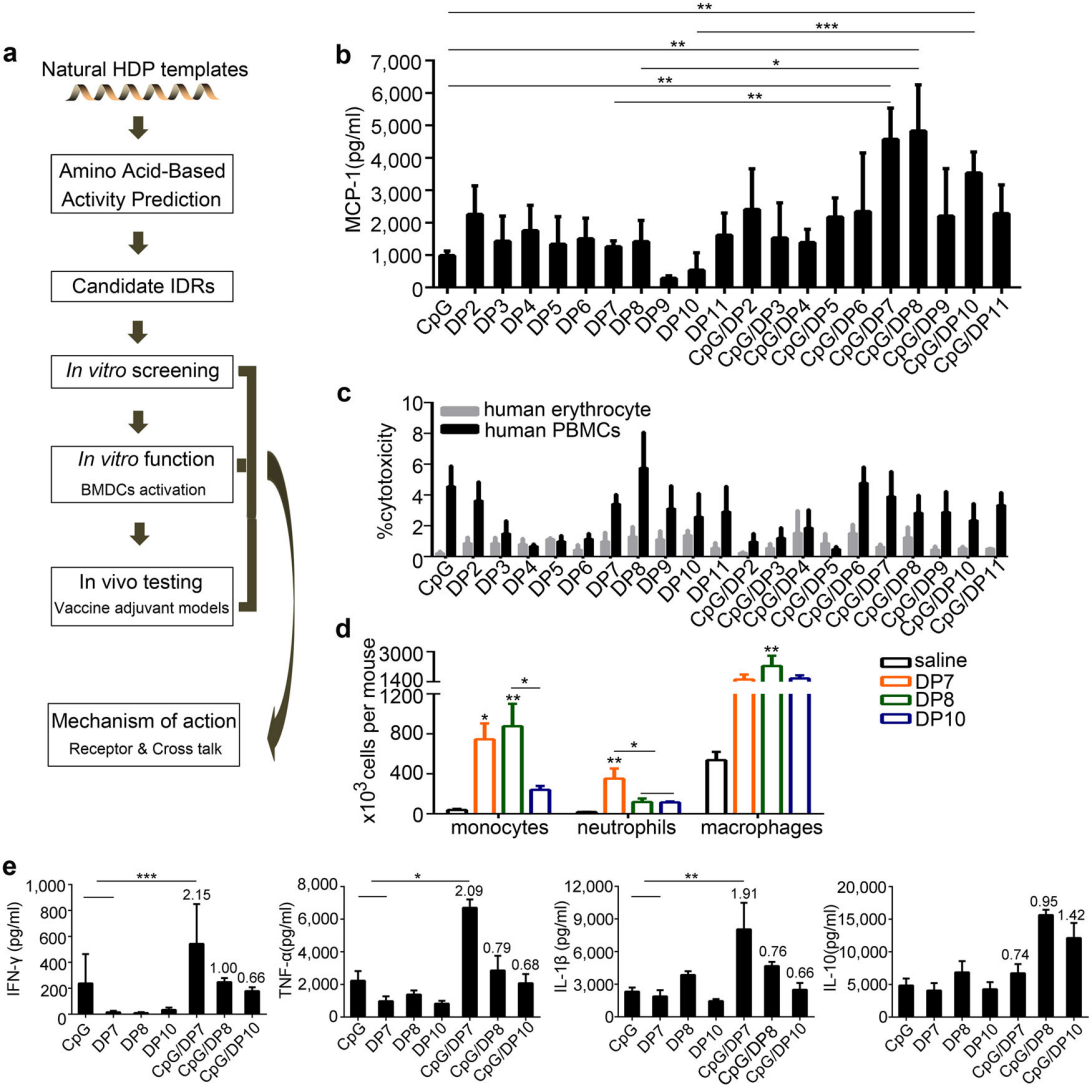
In vitro preliminary screening of defense peptide candidates. **a** A representative flowchart of the IDR design. **b** PBMCs were stimulated with CpG (20 µg/ml), DP2-11 (40 µg/ml), or CpG: DP2-11 (1:2; wt/wt) formulations for 24 h. The secretion of MCP-1 was detected by ELISA. **c** PBMCs or human erythrocytes were incubated with CpG (20 µg/ml), DP2-11 (40 µg/ml), or CpG/DP2-11 (1:2; wt/wt) formulations for 24 h. After that, the supernatants were collected, and LDH release from PBMCs and total hemoglobin release from red blood cells were measured. **d** DP7, DP8, and DP10 induced the significant recruitment of neutrophils (Gr1^+^F4/80^−^), monocytes (F4/80^+^Gr1^+^), and macrophages (F4/80^+^CD11b^+^) to the injection site at 24 h post-injection (*N* = 5/group)**. e** PBMCs were stimulated with CpG (20 µg/ml), DP7 (40 µg/ml), DP8 (40 µg/ml), DP10 (40 µg/ml), CpG/DP7, CpG/DP8 or CpG/DP10 (1:2; wt/wt) formulations for 48 h. The secretion of the indicated cytokines was detected. *N* = 3 per group. Bars represent means and SEM. The numbers in the graph indicate the synergistic values. *P < 0.05; **P < 0.01; ***P < 0.001.

An IDR discovery strategy using computer-aided design and selection was applied in the preparation of a class of IDRs^22^. Through this process, 10 candidate IDRs (DP2-11) were chosen for extensive in vitro analysis (Fig. 1a and Supplementary Table 1). It was previously reported that IDRs were combined with CpG to induce stronger immunostimulatory activities^16,31,32^. In this study, the screening was based on the combination of DPs and CpG.

An agarose electrophoretic mobility shift assay (AEMSA) demonstrated that compared to CpG alone, the coincubation of CpG and DP7/DP8/DP10 caused the formation of a slow migrating aggregate with no discernable dissociation when formed at a 1:2 (wt/wt) ratio of CpG and DP7/DP8/DP10 (Supplementary Fig. 1a). Similarly, the incubation of CpG with the other DPs had the same outcomes (data not shown). The production of MCP-1 serves as a principal marker to monitor the impact of IDRs on immune activation^16^. The results showed that a 1:2 (wt/wt) ratio of CpG to DP2-11 generally favored relatively high MCP-1 secretion with respect to other ratios (Fig. 1b and Supplementary Fig. 1b). In particular, a 1:2 (wt/wt) ratio of CpG to DP7, DP8, and DP10 was a stronger inducer of MCP-1 and produced synergistic effects of 2.04, 2.01, and 2.33, respectively. This synergistic effect was calculated as the total release of chemokines induced by a CpG/DPs complex divided by the additive release of chemokines induced by the individual complex components.

In consideration of safety concerns, the toxicity of the CpG/DP2-11 complexes at various ratios was assessed by examining the release of hemoglobin from red blood cells and lactic dehydrogenase (LDH) from PBMCs. As a result, DP2-11 alone or CpG/DP2-11 complexes caused minimal erythrocyte lysis and had little effect on LDH release from PBMCs (Fig. 1c and Supplementary Fig. 1c). These results demonstrate that the CpG/DP7, CpG/DP8, and CpG/DP10 complexes with a 1:2 ratio (wt/wt) were safe and effective immunomodulatory adjuvant candidates.

C57BL/6 mice (*N* = 5) were intraperitoneally injected with 200 μg DP7, DP8, DP10, or sterile saline, and then the total resident peritoneal cells were lavaged at 24 h post-injection and analyzed by flow cytometry. There was a significant increase in the numbers of monocytes and neutrophils in the peritoneal lavage of the DP7-treated mice; however, there was also a modest but nonsignificant increase in the number of macrophages (Fig. 1d and Supplementary Fig. 8a, b). Cytokine/chemokine induction and immune cell recruitment are important aspects of the immunomodulatory activities shared by many adjuvants^2^. To further select the best candidate adjuvant, the production of cytokines and the recruitment of immune cells were detected. Cytokine profiling showed that a 1:2 ratio (wt/wt) for the CpG/DP7 complex markedly outperformed the same ratio for the CpG/DP8 and CpG/DP10 complexes in inducing the production of the proinflammatory cytokines IFN-γ, TNF-α, and IL-1β while the production of the anti-inflammatory cytokine IL-10 was unaffected by the CpG/DP7 complex (Fig. 1e). These results demonstrate that DP7 was the lead candidate and that the CpG/DP7 complex with a 1:2 ratio was a safe and effective immunomodulatory adjuvant.

### BMDC activation by the CpG/DP7 complex in vitro

**Fig. 2:**
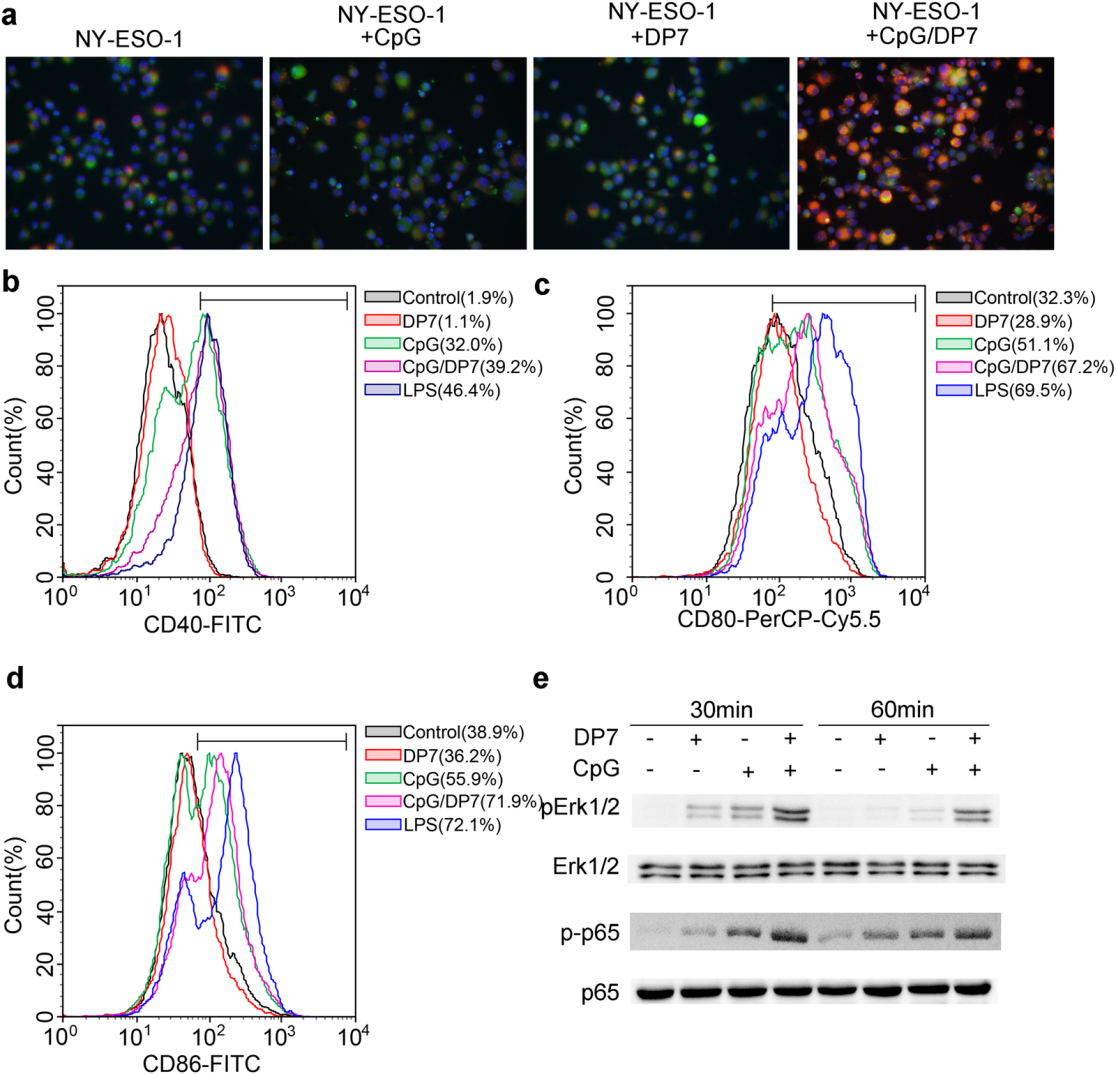
The effect of the CpG/DP7 complex on BMDC activity. **a** NY-ESO-1 was labeled with Alexa Fluor® 594. BMDCs were stimulated with labeled-NY-ESO-1, labeled-NY-ESO-1/CpG, labeled-NY-ESO-1/DP7, or labeled-NY-ESO-1/CpG/DP7 complex for 1 h. Cell membranes tagged with green fluorescent dye. Cells were fixed and stained with DAPI followed by observation with confocal microscopy. **b–d** BMDCs were incubated with CpG, DP7, or CpG/DP7 for 16 h. Then, the surface maturation markers of CD40 (**b**), CD80 (**c**) and CD86 (**d**) in BMDCs were analyzed by flow cytometry. **e** BMDCs were stimulated with CpG, DP7, or the CpG/DP7 complex for 30 or 60 min and then collected to detect the expression of p-Erk1/2 and p-p65 by western blotting.

The data described above illustrate that the CpG/DP7 complex significantly enhances the production of chemokines and positive regulatory cytokines. We next explored whether the CpG/DP7 complex is a potent stimulator of dendritic cell function. Therefore, we analyzed the effect of the CpG/DP7 complex on antigen uptake, cellular maturation, and signaling activation in BMDCs. Compared with that stimulated by CpG or DP7 alone, the uptake of NY-ESO-1 or OVA was enhanced in BMDCs stimulated for 1 h or 4 h by the CpG/DP7 complex (Fig. 2a and Supplementary Fig. 2). Moreover, the CpG/DP7 complex effectively upregulated the expression of the costimulatory molecules CD40, CD80, and CD86 (Fig. 2b–d and Supplementary Fig. 8c). Finally, the CpG/DP7 complex prominently activated the phosphorylation of p-Erk1/2 and p-p65 (Fig. 2e). These findings suggested that the CpG/DP7 complex effectively activated dendritic cell function, playing a crucial role in the adaptive immune response.

### Antitumor immunity induced by alum/CpG/DP7-based vaccines

**Fig. 3:**
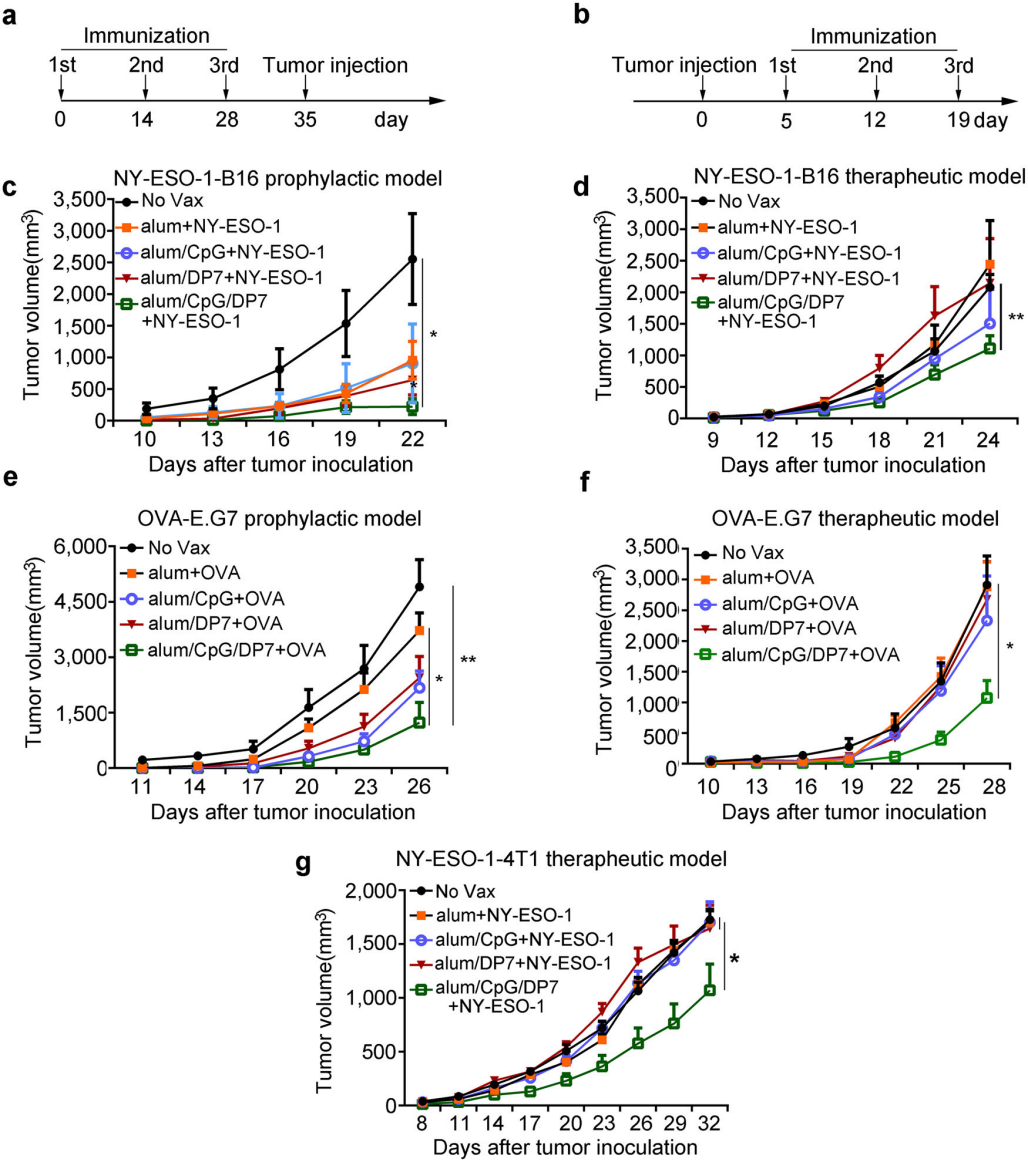
Alum/CpG/DP7-based vaccines suppressed tumor progression in multiple models. **a** Schedules of vaccine immunization in the prophylactic models. In the prophylactic models, mice were treated with the indicated vaccines (on days 0, 14, and 28) and then challenged s.c. with 2 × 10^5^ NY-ESO-1-B16 (**c**) or 2 × 10^6^ E.G7-OVA (**e**) cells on day 35 according to the schedules. The tumor volume was measured every 3 days. **b** Schedules of vaccine immunization in the therapeutic models. In the therapeutic models, mice were challenged s.c. with 2 × 10^5^ NY-ESO-1-B16, (**d**) 2 × 10^6^ E.G7-OVA (**f**), or 2 × 10^5^ NY-ESO-1-4T1 (**g**) cells on day 0 and then treated with the indicated vaccines (on days 5, 12, and 19) according to the schedules. The tumor volume was measured every 3 days. *N* = 10/group. Bars represent means and SEM. *P < 0.05, **P < 0.01, ***P < 0.001.

CpG clearly leads to a potent Th1-type immune response^33^. Thus, alum was formulated with CpG and DP7 to elicit a balanced Th1 and Th2 immune response. NY-ESO-1, a member of cancer/testis family of antigens, is expressed in numerous human cancer types, but with the exception of the testis, it is not expressed in normal tissues^34^. Its ability to elicit spontaneous humoral and cellular immune responses has rendered it a promising candidate antigen for tumor vaccines^35^. Therefore, in addition to the model antigen OVA, NY-ESO-1 was also selected as a vaccine antigen. In prophylactic or therapeutic animal experiments, NY-ESO-1-B16 and OVA-E.G7 tumor-bearing C57BL/6 mice or NY-ESO-1-4T1 tumor-bearing BALB/c mice were used to evaluate the antitumor activity of the alum/CpG/DP7 adjuvant. The vaccine immunization schedules used for the prophylactic and therapeutic models are shown in Fig. 3a, b and in the METHODS database.

The effects of NY-ESO-1-alum/CpG/DP7 vaccine treatment on NY-ESO-1-B16 tumors are shown in Fig. 3c, d. NY-ESO-1-B16 tumor volumes were reduced by 88.5% and 36.3% compared with the No Vax tumor volumes in the prophylactic and therapeutic models, respectively (*P* < 0.05). The powerful antitumor activity of the OVA-alum/CpG/DP7 vaccine was observed in the established prophylactic and therapeutic E.G7-OVA thymoma models (*P* < 0.05) (Fig. 3e, f). Additionally, in a therapeutic NY-ESO-1-4T1 model, the ability of the NY-ESO-1-alum/CpG/DP7 vaccine to delay tumor growth was apparent (Fig. 3g).

To observe the toxicity of alum/CpG/DP7-based vaccines, the health status of mice in the prophylactic animal experiment was evaluated. No significant adverse consequences were observed in terms of gross measures such as weight loss, ruffling of fur, and behavior changes. Furthermore, there were no pathological changes in the heart, liver, lung, spleen, or kidney. The results of the body weights and tissue staining of mice that were treated with NY-ESO-1 combined with the indicated adjuvants are shown in Supplementary Fig. 3.

To evaluate the effect of the indicated adjuvants on blood cells, a routine blood examination was carried out. Mice were immunized with NY-ESO-1 plus the indicated adjuvants on day 0, 14, and 28. One week after the third immunization, White blood cell count (WBC), red blood cell count (RBC), Platelet (PLT) count, hemoglobin (HGB) level, hematocrit (HCT), mean platelet volume (MPV), mean corpuscular hemoglobin (MCH), mean corpuscular hemoglobin concentration (MCHC), and mean corpuscular volume (MCV) were determined. Compared to No vax group, the level of WBC in CpG and alum/CpG/DP7 were enhanced. However, most of the biochemical indicators did not differ significantly between each group. The results suggested that adjuvants did not cause obvious adverse effects in blood cells (Supplementary Fig. 4).

In order to investigate the effect of the adjuvants on the functions of major organs, a series of serum biomarkers indicating the physiological function of these organs were measured. Liver function indicators included total protein (TP), albumin (ALB), alanine aminotransferase (ALT), aspartate transaminase (AST), alkaline phosphatase (ALP), glucose (GLU), low-density lipoprotein cholesterol (LDL-C), and high-density lipoprotein cholesterol (HDL-C). Kidney function indicators included creatinine (CREA), uric acid (UA), and urea (UREA). Heart function indicators included actate dehydrogenase (LDH). The results showed that no obvious differences were found between the indicated adjuvants-treated groups and the No vax group (Supplementary Fig. 5). These data indicated that the alum/CpG/DP7 adjuvant showed safe and promising effects against tumor growth. Also, the results of hematological studies and the measure of serum biochemical biomarkers proved the CpG/DP7 complex as a safe and effective immunomodulatory adjuvant.

### Enhanced humoral immunity and cellular immunity induced by alum/CpG/DP7-based vaccines

**Fig. 4:**
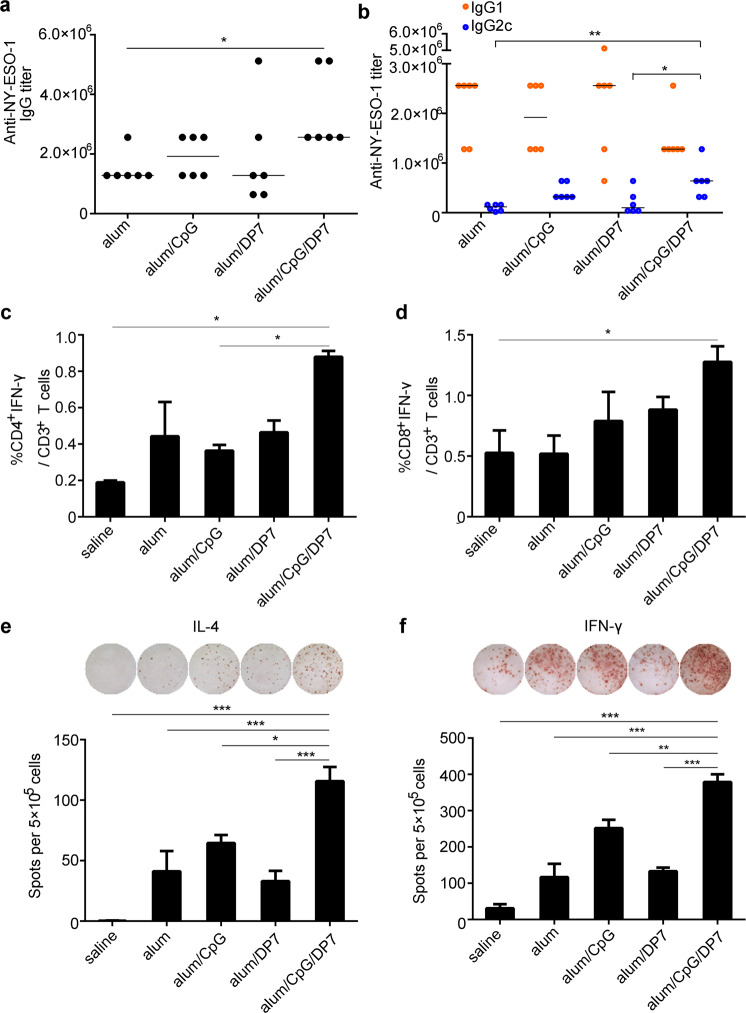
Enhancement of humoral immunity and the Th1/Th2 balance were induced by alum/CpG/DP7-based vaccines. **a**, **b** Mice (*N* = 6/group) were immunized with NY-ESO-1 plus various adjuvants on days 0, 14, and 28. The levels of IgG (**a**), IgG1 and IgG2c (**b**) specific for NY-ESO-1 were detected by ELISA one week after the third immunization. Bars represent median. **c**, **d** The frequencies of IFN-γ-secreting CD4^+^ and CD8^+^ T cells were examined by intracellular IFN-γ staining (*N* = 3/group). **e**, **f** The average number of IL-4/IFN-γ-secreting splenocytes was calculated (*N* = 3/group). Bars represent means and SEM. *P < 0.05, **P < 0.01, ***P < 0.001.

The ability of alum/CpG/DP7-based vaccines to elicit humoral immunity was detected by immunization of mice with NY-ESO-1 plus various adjuvants and the measurement of NY-ESO-1-specific IgG, IgG1, and IgG2c. The NY-ESO-1-alum/CpG/DP7 vaccine prominently outperformed the other three vaccines in boosting the anti-NY-ESO-1 IgG titer (Fig. 4a). The NY-ESO-1-alum/CpG/DP7 vaccine promoted isotype switching by elevating the anti-NY-ESO-1 IgG2c titer, leading to an enhanced Th1/Th2 balance (Fig. 4b).

Activated T lymphocytes make prominent contributions to antitumor immunity. We thus explored whether the activation of IFN-γ-secreting CD4^+^ or CD8^+^ T cells was the consequence of the effects of the NY-ESO-1-alum/CpG/DP7 vaccine. Mice (*N* = 3) were immunized with NY-ESO-1 plus the above-described adjuvants on days 0, 14, and 28, and then splenocytes were isolated one week after the third immunization for flow cytometry analysis. The percentages of IFN-γ-producing CD4^+^ or CD8^+^ effector T cells in the alum, alum/CpG, and alum/DP7 groups were modestly higher than those in the No Vax group. However, upon stimulation with the NY-ESO-1-alum/CpG/DP7 vaccine, the highest proportion of IFN-γ-producing CD4^+^ or CD8^+^ effector T cells was observed (Fig. 4c, d and Supplementary Fig. 8d, e). Similarly, the largest number of spot-forming cells (SFCs) for IFN-γ and IL-4 was induced by the NY-ESO-1-alum/CpG/DP7 vaccine (Fig. 4e, f). These data indicated that the alum/CpG/DP7 adjuvant induces promising humoral and cellular immunity.

### Identifying the molecular mechanism of DP7

**Fig. 5:**
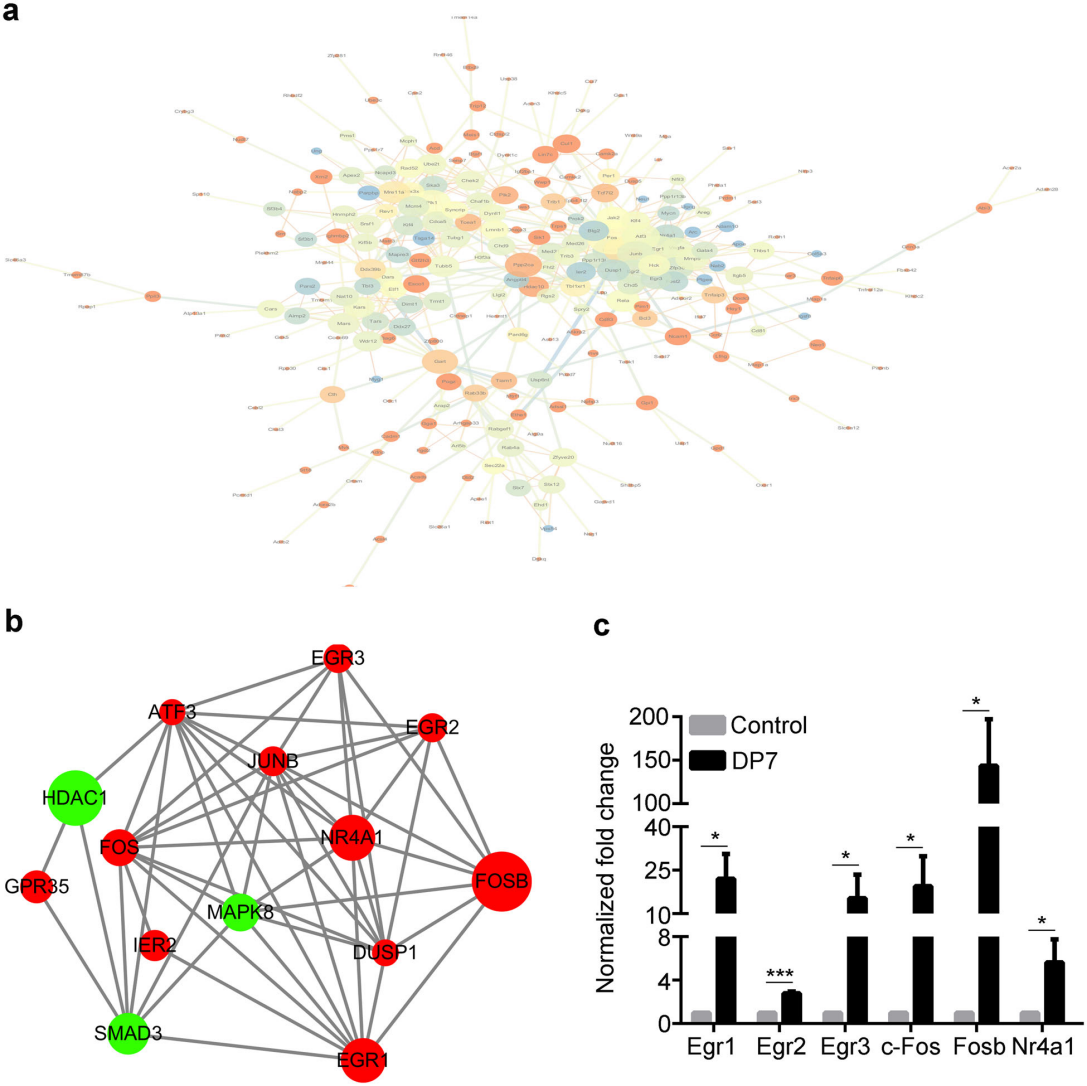
Signaling pathways involved in peptide DP7 activity in BMDCs. **a** An Egr1-centered protein–protein association network of the 117 genes, as analyzed by STRING. **b** An Egr1-centered protein-protein association network of 6 genes involved in the Egr1-centered network. **c** JAWSII cells were stimulated with DP7 (40 μg/ml) for 30 min and then harvested for RT-qPCR analysis. The control indicates the nonstimulated group. Data are representative of three experiments with three replicates each. Statistical analyses were done using the t-test. Bars represent means and SEM. *P < 0.05, ***P < 0.001.

An explicit description of the mechanism of action of an adjuvant is essential for vaccine and adjuvant developers^36^. To better understand the involvement of signaling pathways, RNA-Seq analysis was employed to determine the change in gene expression of BMDCs upon challenge with peptide DP7 for 30 min. Of the 22,956 detected genes, We identified a total of 45 upregulated and 72 downregulated genes in BMDCs with DP7 stimulation compared with the control cells. So there is 117 genes in total. To understand the cellular effects of these differentially expressed gene transcripts, we performed protein association analysis using STRING, an online functional protein-protein association tool used for a large number of organisms. An *Egr1-*centered transcription factor network was detected (Fig. 5a, b). We hypothesized that *Egr1*, *Egr2*, *Egr3, c-Fos*, *Fosb,* and *Nr4a1* could contribute to the activity of DP7, enhancing the immune regulatory function of BMDCs. The elevated expression of *Egr1, Egr2, Egr3, c-Fos, Fosb,* and *Nr4a1* mRNA were confirmed in vitro by RT-qPCR (Fig. 5c), which was in line with the results of RNA-seq analysis (Supplementary Table 2).

### Activation of the Gi protein-coupled/ErK pathway by DP7

**Fig. 6:**
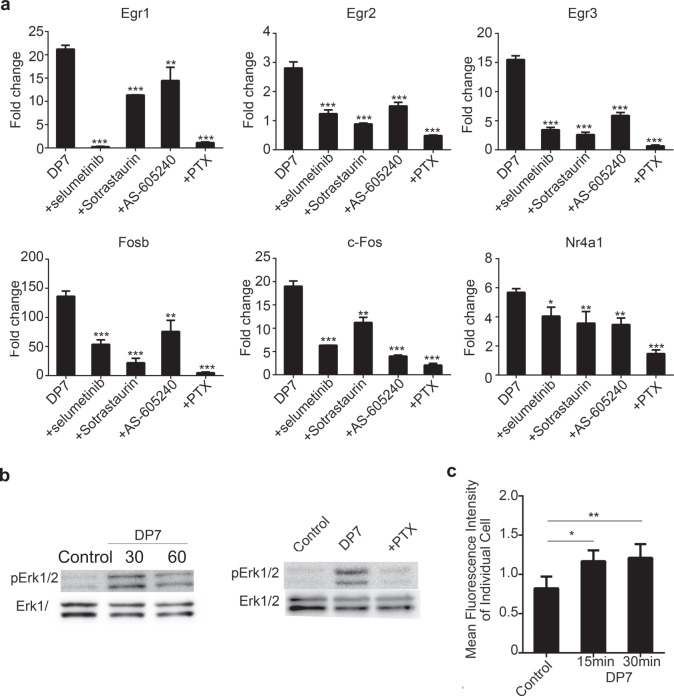
Signaling pathways involved in DP7 activity. **a** JAWSII cells were pretreated with chemical inhibitors, stimulated with DP7 (40 μg/ml) for 30 min, and then harvested for RT-qPCR analysis. Data are representative of three experiments with three replicates each. Statistical analyses were done using the t-test. Bars represent means + SEM. **b** JAWSII cells were pretreated with PTX and then stimulated with the peptide DP7 for 30 and 60 min. Erk1/2 phosphorylation was detected by western blotting. **c** JAWSII cells were stimulated with the peptide DP7 for 15 and 30 min. Intracellular Ca^2+^ levels were measured with a Celligo® image cytometer; control indicates the nonstimulated group. Bars represent means and SEM. *P < 0.05, **P < 0.01, ***P < 0.001.

Small molecule inhibitors were employed to elucidate the upstream signaling pathways involved in the expression of 6 transcription factors: selumetinib (MEK1/2 inhibitor), BIBR796 (p38α inhibitor), sp600125 (pan JNK inhibitor), XMD8-92 (Erk5 inhibitor), sotrastaurin (PKCθ inhibitor), AS-605240 (PI3Kγ inhibitor), LY294002 (PI3Kα/β/γ inhibitor) and pertussis toxin (PTX, Gi protein inhibitor). BMDCs were pretreated with 10 μM selumetinib, 5 μM BIBR796, 10 μM sp600125, 10 μM XMD8-92, 10 μM sotrastaurin, 10 μM AS-605240, and 10 μM LY294002 at 37 °C for 1 h and with 200 ng/ml PTX at 37 °C for 3 h. BMDCs were exposed to DP7 at 37 °C for 30 min, and then the mRNA expression of the transcription factors was examined by RT-qPCR. Compared to that in the presence of DP7 alone, the mRNA expression of transcription factors was significantly suppressed in the presence of selumetinib, sotrastaurin, AS-605240, and PTX (Fig. 6a). However, BIBR796, sp600125, XMD8-92, and LY294002 showed little or no inhibitory effect on the mRNA expression of transcription factors (Supplementary Fig. 6).

Moreover, DP7 resulted in the enhancement of Erk1/2 phosphorylation (Fig. 6b) and the elevation of intracellular Ca^2+^ levels (Fig. 6c), which are two important properties of Gi protein activation^37^. Similar to the peptide LL-37^38^, the chemical inhibitor PTX partially abolished the activation of pErk1/2 by the peptide DP7 (Fig. 6b). The above results demonstrated that PTX abolished the mRNA expression of transcriptional factors and the activation of pErk1/2, suggesting the involvement of a Gi-coupled receptor in DP7 signaling.

### Identification of GPR35 as a potential interaction partner for DP7

**Fig. 7:**
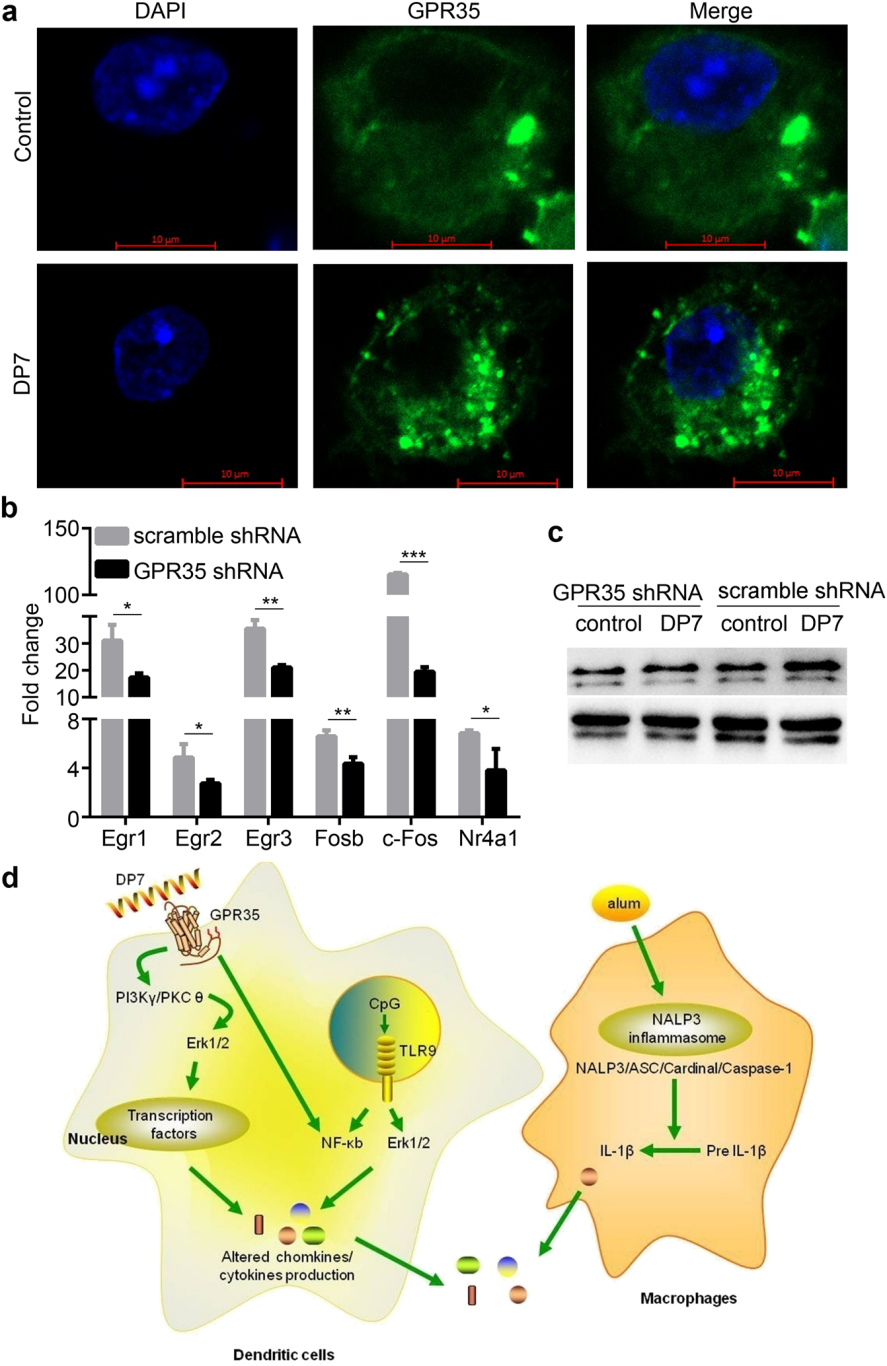
GPR35 was essential for the activity of the peptide DP7. **a** Representative fluorescence images of GPR35 internalization induced by stimulation with DP7 (40 μg/ml) for 30 min in BMDCs. Control indicates the nonstimulated group. **b**, **c** GPR35 was inhibited by transfection of JAWSII cells with an shRNA lentivirus, which were then selected with puromycin for 5 days. After stimulation with the peptide DP7 (40 μg/ml) for 30 min, cells were lysed for the detection of transcription factor expression by RT-qPCR (Data are representative of three experiments with three replicates each; Statistical analyses were done using the t-test.) (**b**) or Erk1/2 phosphorylation by western blotting (**c**). Control indicates the nonstimulated group. **d** A simplified schematic of the mechanisms of action of alum/CpG/DP7 in dendritic cells and macrophages. Bars represent means and SEM. *P < 0.05, **P < 0.01, ***P < 0.001.

Surprisingly, two G protein-coupled receptors were found among the upregulated genes by RNA-seq analysis: GPR52 and GPR35 (Supplementary Table 2). GPR52 is a Gs protein-coupled receptor^39,40^, whereas GPR35 is a Gi/o protein-coupled receptor^41,42^. Therefore, we speculated that GPR35 was involved in DP7 signaling. Receptor internalization is regarded as a hallmark feature of high-efficacy ligands involved in the activation of most GPCRs^43^. Immunofluorescence staining of BMDCs revealed that GPR35 in the untreated cells was principally located at the cell membrane. DP7 stimulation resulted in the internalization of GPR35 into the cytoplasm (Fig. 7a), and similar internalization was observed in JASWII and RAW264.7 cells (Supplementary Fig. 7).

To examine whether GPR35 was the potential interacting partner of the peptide DP7, a 70% reduction in GPR35 mRNA expression was achieved by transfection of JAWSII cells with a GPR35 shRNA-encoding lentivirus (data not shown). The results showed that significant downregulation of the 6 transcription factors was detected after the partial knockdown of GPR35 by shRNA (Fig. 7b). Moreover, GPR35 knockdown partially suppressed the phosphorylation of Erk1/2, which was activated by the peptide DP7 (Fig. 7c). These results suggested that GPR35 was a potential interacting protein partner for the peptide DP7.

According to our results and previous reports, the mechanisms of action of alum/CpG/DP7 were described (Fig. 7d). The peptide DP7 partially interacts with GPR35 on the cell surface, which activates the PI3Kγ, PKCθ and Erk1/2 signaling pathways followed by the activation of transcription factors. Transcription factors regulate the production of chemokines or cytokines. Alternatively, the activation of NF-κB by the peptide DP7 may affect the production of chemokines or cytokines. The binding of CpG to TLR9 induces the activation of Erk1/2 or NF-κB, resulting in the release of inflammatory factors such as IL-6 and TNF-α. Alum activates the NALP3 inflammasome, which regulates the cleavage and secretion of the proinflammatory cytokines IL-1β and IL-18^44^. The expression of chemokines or cytokines induced by alum, CpG, and the peptide DP7 is responsible for the enhanced immune response.

## Discussion

Their wide distribution and key immunomodulatory effects on the innate immune response have made HDPs potential candidates that may serve as effective and promising vaccine adjuvants^11^. The immense structural diversity and promising clinical significance of HDPs provide enormous templates and opportunities for the design of IDRs^45^. Designing and screening effective immunomodulatory peptides and identifying interacting partners are the two main challenges for the development of IDRs^46^. Here, a platform was described in which we successfully designed and measured the innate defense regulator peptide DP7, a possible ligand of GPR35 that exhibited strong immunomodulatory activity and acted as an effective adjuvant in formulations with CpG and alum. Given the feasibility of this approach and the diversity of HDPs, we believe that the platform is applicable to HDPs other than Bac2A for designing effective IDRs.

Our in vitro screening method is based on evaluating the production of MCP-1, IFN-γ, TNF-α, and IL-1β. MCP-1 acts as a vital indicator of immunomodulatory activity by HDPs and IDRs^15,30,47^, which are potent chemoattractants for monocytes/macrophages, natural killer (NK) cells, and neutrophils and have antitumor activity^48^. Our data indicated that DP7, DP8, and DP10 combined with CpG resulted in improved efficacy in the production of MCP-1 compared with other defense peptides. Moreover, compared with DP8 and DP10, DP7 combined with CpG significantly stimulated the secretion of IFN-γ, which is essential for activating NK cells, enhancing antigen presentation, inducing Th1 immune responses and stimulating antitumor immunity^49^. CpG, a Toll-like receptor 9 agonist, initiates Th1-biased immunity and acts as a prospective clinical vaccine adjuvant^50,51^. The synergistic effect of IDRs and CpG on the immune response has been studied, but the knowledge of the effects is limited^17,20,52^.

Adjuvants modulate the immune response and enhance vaccine activity mainly by directly or indirectly influencing antigen presentation by dendritic cells^53^. DP7 combined with CpG effectively promoted antigen uptake and upregulated the costimulatory molecules CD40, CD80, and CD86. Therefore, based on the above studies, we speculate that combined DP7 and CpG formulations would be effective adjuvants for enhancing antigen-specific immune responses. Alum-containing adjuvants have been mostly used in licensed human vaccines with high levels of production due to their safety and efficacy^54^. Moreover, the polarized Th2-type immune response elicited by alum^55^ suggests that its combination with the DP7/CpG formulation maybe results in a Th1/Th2 balance.

In animal tumor models, vaccines with an alum/DP7/CpG compound adjuvant significantly suppressed tumor growth. Additionally, the alum/DP7/CpG adjuvant induced a significant humoral immune response and led to an enhanced Th1/Th2 balance by the increased IgG2c titer. The activation of IFN-γ-producing type 1 T helper cells and cytotoxic T lymphocytes plays an essential role in cancer therapy and is a promising strategy for the development of modern adjuvants^56^. Vaccine treatment with the alum/DP7/CpG compound adjuvant notably activated IFNγ-producing CD4^+^/CD8^+^ T cells and even memory CD4^+^ T cells. These effects resulted in suppressed tumor growth in multiple tumor models, enhanced humoral immunity and strengthened T cell immune responses, all of which demonstrates the promise of the platform for IDR- or IDR-based adjuvants.

Previous studies have simply outlined the common mechanisms of action of HDPs and IDRs. Briefly, HDPs and IDRs interact with G_i_ protein-coupled receptors in the cell membrane or intracellular receptors, leading to the induction of signal transduction pathways (MAPK, PI3K, NF-κB, Src, and others) followed by the activation of transcription factors (NF-κB, AP-1, AP-2, EGR, and others)^57^. Consistent with the similarities to the mechanism of action, the RNA sequencing analysis indicated the notably enhanced transcription of GPR35 under DP7 stimulation. GPR35 is a G_i_ protein-coupled receptor with high expression in mouse neutrophils, monocytes, T cells, and dendritic cells^58^, playing an important role in leukocyte recruitment^59^ and possibly emerging as a drug target^60^. Moreover, receptor internalization and elevated cytosolic calcium levels, which are two typical physiological effects of GPR35 activation^61,62^, were clearly visualized in dendritic cells after DP7 challenge. DP7 specifically activates the ERK1/2 signaling pathway, which is a hallmark of GPR35 activation^37^; this results in the activation of the transcription factors EGRs, c-Fos, and FosB, which contribute to the effector functions of HDPs and IDRs^57^. However, the activation of ERK1/2 and transcription factors was substantially suppressed by the knockdown of GPR35 in the presence of DP7, which confirmed the involvement of GPR35 in DP7 signaling.

We demonstrate that our stepwise approach for adjuvant design provides a platform that can be applied to rationally design effective IDR adjuvants in vaccines that will enhance the antigen-specific immune response. The design strategy and technologies used to examine the mechanism of action to address the efficacy and safety requirements of vaccine adjuvants should have applications beyond their use in IDRs with Bac2A as a template.

## Methods

### Mice and cell lines

Female 6- to 8-week-old C57BL/6 and BALB/c mice (Beijing HuaFukang Biological Technology Company, Beijing, China) were maintained in Specific Pathogen Free conditions. All animal experiments and protocols used in this study were approved by the Ethics Review Committee for Animal Experimentation of Sichuan University. B16-F10 melanoma cells, 4T1 breast cancer cells, OVA-transfected OVA-E.G7 T lymphoma cells, and JAWSII cells were obtained from American Type Culture Collection (ATCC, Manassas, VA) and cultured according to the recommended guidelines of ATCC. B16-F10 cells stably expressing NY-ESO-1 (NY-ESO-1-B16) and 4T1 monoclonal cell line stably expressing NY-ESO-1 (NY-ESO-1-4T1) were generated by limit-dilution cloning, which were confirmed by RT-PCR and Western blotting.

### Reagents

DP2-11 was synthesized by using fluorenyl-methyloxycarbonyl (Fmoc) chemistry at the Shanghai Science Peptide Biological Technology Co., Ltd. (Shanghai, China). The synthesized peptides were purified by high-performance liquid chromatography to obtain 95% purity, and their molecular weights were confirmed by mass spectrometry. The peptide powder was reconstituted in sterile water at a concentration of 10 mg/ml and stored at −20 °C. CpG ODN (murine TLR9 agonist, 5′-tccatgacgttcctgacgtt-3′) contains a full phosphorothioate backbone and was synthesized by Invitrogen Life Technologies. The CpG ODN powder was dissolved in sterile water at a concentration of 5 mg/ml and then stored at −20 °C until use. The aluminum hydroxide gel adjuvant (alum) was obtained from Brenntag Biosector, Frederikssund, Denmark. The NY-ESO-1 antigen, in which the endotoxin level was approximately 0.02 EU/μg, was purified via four process steps including Ni-chelating Sepharose affinity chromatography (GE Healthcare, Piscataway, NJ), excision of the Trx-His6-tag, removal of the Trx-His6-tag with second Ni-chelating Sepharose affinity chromatography, and Q-ion-exchange chromatography (GE Healthcare, Piscataway, NJ). Endotoxin-free ovalbumin (OVA) was purchased from InvivoGen.

### Agarose electrophoretic mobility shift assays (AEMSA)

A total of 5 μg CpG ODN was incubated with various doses of DP7 in a final volume of 50 μL PBS (pH 7.4) in 37 °C for 10–15 min. The reactions were stopped by the addition of 10 µl of 6x DNA loading dye and immediately subjected to electrophoresis in a 1% agarose gel at 90 V for 20 min. The DNA was visualized by a gel imaging system.

### Isolation of human peripheral blood mononuclear cells (PBMCs) and mouse bone marrow-derived dendritic cells (BMDCs)

Human peripheral blood was collected from healthy donors. PBMCs were isolated using density-gradient centrifugation with Ficoll-Paque PLUS (GE Healthcare, Milwaukee, WI, USA) for 20 min at 1000 g. The PBMC fractions were collected and then transferred to a new 15 ml centrifugation tube. The PBMCs were washed twice with RPMI 1640. Finally, the PBMCs were resuspended at an appropriate density in RPMI 1640 supplemented with 10% FBS for further experiments.

Bone marrow cells were isolated from C57BL/6 mice and cultured in the presence of 10 ng/ml recombinant murine GM-CSF and IL-4 (Pepro Tech, London, UK). BMDCs from 5-day-old cultures were collected and/or enriched with CD11c MicroBeads (Miltenyi Biotec GmbH, Bergisch Gladbach, Germany) for further experiments.

### Chemokine and cytokine release

For the formulation of the adjuvant combination, 10 μg CpG ODN was incubated with various doses of DP2-11 in a final volume of 50 μL RPMI 1640 at 37 °C for 10–15 min to form CpG/DP2-11 complexes at various ratios (4:1–1:4; wt/wt). To analyze chemokine production, PBMCs were seeded in 48-well plates at a density of 1 × 10^6^ cells/ml (500 µl), incubated at 37 °C for 1 h and then stimulated with CpG, DP2-11, and CpG/DP2-11 formulations with various ratios. After 24 h of incubation at 37 °C, the supernatants were collected and then analyzed by ELISA (R&D System, Minneapolis, MN, USA) to detect MCP-1 release. Briefly, 200 μl of each sample was added into per well of MCP-1 microplate and incubated for 2 h at room temperature. After the wells were washed three times, the plate was incubated with 200 μl of MCP-1 conjugate for 1 h at room temperature. After the wells were washed five times, 200 μl of Substrate Solution was added to per well for 30 min at room temperature. The reactions were stopped by adding 50 μl of stop solution, and the optical density was determined using a microplate reader set to 450 nm.

To further examine the effect of DP7, DP8, and DP10 on cytokine production, PBMCs were seeded in 48-well plates at a density of 4 × 10^6^ cells/ml (125 µl), incubated at 37°C for 1 h and then exposed to CpG (20 µg/ml), DP7, DP8, or DP10 (40 µg/ml) or a 1:2 (wt/wt) ratio of CpG/DP7, CpG/DP8 or CpG/DP10 for 48 h, followed by the detection of IFN-γ, TNF-α, IL-1β, and IL-10 secretion using a Milliplex MAP Kit (HCYTOMAG-60K, Millipore, USA).

### Cytotoxicity assay

The toxic effect of the CpG/DP2-11 combination was assayed by measuring lactate dehydrogenase (LDH) release from PBMCs and hemoglobin release from human red blood cells. For the formulation of the adjuvant combination, 10 μg CpG ODN was incubated with various doses of DP2-11 in a final volume of 50 μL RPMI 1640 at 37 °C for 10–15 min to form CpG/DP2-11 complexes with various ratios (4:1–1:4; wt/wt).

PBMCs were seeded in 24-well plates at a density of 1 × 10^7^ cells/ml (500 µl), incubated at 37 °C for 1 h, and then stimulated with CpG, DP2-11, and CpG/DP2-11 formulations with various ratios. After 24 h, the LDH enzyme released from cells was quantified by an LDH assay kit. The absorbance of the supernatant was measured using a microplate reader set to 490 nm.

To detect the release of hemoglobin, human red blood cells were washed three times with sterile saline and centrifuged at 1500 rpm for 10 min. The red blood cells were diluted 3-fold in saline, and 100 µL of the cell suspension was plated in 96-well plates followed by stimulation with CpG (20 µg/ml), DP2-11 (40 µg/ml), and the CpG/DP2-11 combination at 37 °C for 24 h. Triton X-100 (1%) was used as a positive control, and sterile saline was considered a negative control. Hemoglobin release was monitored at 450 nm using an ELISA plate reader.

### Leukocyte recruitment

C57BL/6 mice (*N* = 5) were intraperitoneally injected with 200 μg DP7, DP8, or DP10. An equal volume of sterile saline served as a control. Cells in the peritoneal lavage were collected and counted at 24 h postinjection. To analyze the recruitment of leukocytes, cells were stained with PE-anti-mouse-GR-1(clone RB6-8C5; BD Biosciences Cat# 553128), APC-anti-mouse-F4/80 (clone T45-2342; BD Biosciences Cat# 566787) or PE-anti-CD11b (clone M1/70; BD Biosciences Cat# 557397) at 4 °C for 30 min and then detected using a BD FACSCalibur (BD Biosciences, San Jose, CA). Neutrophils, inflammatory monocytes and macrophages were gated as Gr1^+^F4/80^−^, F4/80^+^Gr1^+^, and F4/80^+^CD11b^+^ cells, respectively.

### Analysis of antigen uptake and BMDC maturation

NY-ESO-1 was labeled with Alexa Fluor® 594 with the Microscale Protein Labeling Kit (Invitrogen, Carlsbad, CA, USA). The labeled NY-ESO-1 was incubated with DP7 (40 µg/ml), CpG (20 µg/ml), or the CpG/DP7 combination for 10 min and then incubated with a culture of BMDCs for 1 h. Cell membranes tagged with green fluorescent dye. BMDCs were washed three times with PBS, fixed in 4% paraformaldehyde, and stained with DAPI. Finally, the BMDCs were observed under a confocal laser scanning microscope.

To study the effect of the CpG/DP7 complex on BMDCs, BMDCs were incubated with DP7 (40 µg/ml), CpG (20 µg/ml) or the CpG/DP7 combination for 16 h, stained with APC-anti-Mouse CD11c (Clone HL3; BD Biosciences Cat# 550261), FITC-anti-mouse-CD40 (Clone 3/23; BD Biosciences Cat# 561845), PerCP-Cy5.5-anti-mouse-CD80(clone 16-10A1; BD Biosciences Cat# 560526) or FITC-anti-mouse-CD86 (clone GL1; BD Biosciences Cat# 561962). BMDCs were gated as CD11c^+^ and analyzed for CD40, CD80, CD86 expression by NovoExpress software (ACEA Biosciences, Inc.).

### Western blotting

Protein extracts from each sample were separated by 12% SDS-PAGE and transferred onto polyvinylidene fluoride membranes (Millipore, Billerica, MA). The membranes were blocked with 5% non-fat milk. Then, the protein bands were probed with primary antibodies against NF-κB p65 XP® Rabbit mAb (clone D14E12; Cell Signaling Technology Cat#8242), Phospho-NF-κB p65 (Ser536) Rabbit mAb (clone 93H1; Cell Signaling Technology Cat#3033), Erk1/2 Rabbit mAb (clone 137F5; Cell Signaling Technology Cat#4695) and Phospho- Erk1/2 (Thr202/Tyr204) XP® Rabbit mAb (clone D13.14.4E; Cell Signaling Technology Cat#4370) overnight at 4 °C. Then the membranes were incubated with horseradish peroxidase-conjugated goat anti-rabbit IgG (1:3000; Cell Signaling Technology Cat#7074). The blots derive from the same experiment were processed in parallel. The full, uncropped blot with molecular weight marker are shown in Supplementary Fig. 9.

### Measurement of antibody titers

The levels of NY-ESO-1-specific IgG, IgG1, and IgG2c seven days after the third immunization were evaluated using ELISA. Briefly, 2-fold diluted serum was analyzed on 96-well plates (Nunclon, Roskilde, Denmark) coated with 0.1 μg NY-ESO-1 protein per well. NY-ESO-1-specific antibodies were probed with Goat Anti-Mouse IgG Human ads-HRP (SourhernBiotech Cat#1030-05), Goat Anti-Mouse IgG1 Human ads-HRP (SourhernBiotech Cat#1070-05), and Goat Anti-Mouse IgG2c Human ads-HRP (SourhernBiotech Cat#1079-05). Finally, plates were read on an ELISA reader at a wavelength of 450 nm.

### IFN-γ intracellular staining and ELISpot assay

For IFN-γ intracellular staining, mouse splenocytes were isolated one week not the two week after the third immunization, stimulated with 10 µg/ml NY-ESO-1 for 1 h, and then incubated with Golgi Plug (BD Biosciences, San Jose, CA) for an additional 6 h at 37 °C. Afterward, the cells were stained with FITC-anti-mouse-CD3ε (clone 145-2C11; BD Biosciences Cat# 553062), PE-Cy7-anti-mouse CD8α (clone 53-6.7; BD Biosciences Cat# 552877) or PE-Cy7-anti-mouse CD4 (clone RM4-5; BD Biosciences Cat# 552775) antibodies at 4 °C for 30 min, permeabilized and fixed using a BD Cytofix/Cytoperm kit according to the manufacturer’s protocol. Thereafter, the cells were stained with PE- anti-mouse IFN-γ (clone XMG1.2; BD Biosciences Cat# 554412). T cells in mouse splenocytes were gated as CD3ε^+^. CD4^+^ IFN-γ^+^ or CD8^+^ IFN-γ^+^ were gated from CD3ε^+^ cells to analyze the IFN-γ expression by NovoExpress software (ACEA Biosciences, Inc.).

A mouse IFN-γ-precoated ELISPOT kit and a mouse IL-4-precoated ELISPOT kit (Beijing Dakewei Biotech Company, Beijing, China) were used for the ELISpot assays. Briefly, 5×10^5^ splenocytes were seeded onto mouse IFN-γ-specific monoclonal antibody- and IL-4-specific polyclonal antibody-precoated microplates and then incubated with 10 μg/ml NY-ESO-1 at 37 °C for 24 h. Next, an enzyme-linked colorimetric assay was carried out for further detection. The viable cells were quantified using an ELISpot reader system (Beijing Dakewei Biotech Company, Beijing, China).

### Quantitative real-time PCR(RT-qPCR)

Total RNA was isolated using TRIzol reagent (Takara, Otsu, Japan), further purified by isopropanol/ethanol precipitation and finally dissolved in RNase-Free H_2_O. cDNA was prepared with 5× All-In-One MasterMix with the AccuRT Genomic DNA Removal Kit (Abm Canada Inc., Milton, ON, Canada) and employed as a template to assess the expression of various genes with EvaGreen 2× qPCR MasterMix (Abm Canada Inc., Milton, ON, Canada). The results were analyzed according to the relative expression normalized against β-actin.

### Calcium measurement

JAWSII cells (1 × 10^5^) were seeded into 96-well clear–bottom black plates (Corning, New York, USA.) with six replicate wells per condition and cultured at 37 °C overnight. The cells were washed once with Hank’s buffer followed by incubation with the calcium fluorescence probe Fluo 3-AM (5 μM; excitation at 506 nm and emission at 526 nm) (Beyotime, Hangzhou, China) in Hank’s buffer at 37 °C for 30 min. After treatment, the cells were washed once with Hank’s buffer and subsequently stimulated with DP7 (40 µg/ml) for 15 min or 30 min. Fluorescence was measured by a Celligo® imaging cytometer. The Ca^2+^ levels are represented by the mean fluorescence intensity of individual cells.

### Receptor internalization assays

BMDCs (1 × 10^5^) were plated on a microscope cover glass and cultured at 37 °C overnight. The next day, the cells were stimulated with 40 μg/ml DP7 at 37 °C for 30 min and then fixed in 4% paraformaldehyde in PBS at room temperature for 15 min. After washing with PBS 3 times, the BMDCs were blocked and permeabilized in buffer containing 0.1% bovine serum albumin (BSA), 0.5% Triton X-100, and 4% goat serum in PBS for 1 h. Afterward, the BMDCs were incubated with goat anti-GPR35 (clone M-14; Santa Cruz Biotechnology Cat# sc-79507; 1:100) in 3% BSA/PBS buffer at 4 °C for 16 h, followed by staining with the FITC-labeled anti-goat IgG (H + L) secondary antibody (1:100, ZSGB-BIO, Beijing, China) in 3% BSA/PBS buffer at 37 °C for 1 h. BMDCs were finally stained with DAPI and stored at 4 °C until imaging. Confocal imaging was performed with an LSM 800 Zeiss confocal microscope.

### mRNA sequencing by Illumina HiSeq

The total RNA of tumor samples was extracted using TRIzol Reagent (Invitrogen, USA)/RNeasy Mini Kit (Qiagen, USA). The quantity of total RNA was measured by a Qubit® RNA Assay Kit in a Qubit® 2.0 Fluorometer (Thermo Fisher Scientific Inc, USA). The RNA integrity was assessed by an RNA Nano 6000 Assay Kit (Agilent Technologies, USA). The sequencing libraries were constructed from amplified RNA (1 μg) from each sample according to the manufacturer’s protocol (NEBNext® Ultra™ RNA Library Prep Kit for Illumina®, USA) and sequenced on an Illumina HiSeq instrument according to the manufacturer’s instructions (Illumina, USA).

### GPR35 silencing by lenti-shRNA

To construct the GPR35 shRNA vector, the GPR35 shRNA primers 5’CCGGCCTGGATGCCATCTGTTACTACTCGAGTAGTAACAGATGGCATCCAGGTTTTT 3’ and 5’ AATTAAAAACCTGGATGCCATCTGTTACTACTCGAGTAGTAACAGATGGCATCCAGG3 were annealed and ligated into the pLKO.1-TRC vector. pLKO.1-Scrambled shRNA was employed as a negative control. Lentiviral particles were produced by transfection of 293 T cells with the pLKO.1-shRNA vector in combination with the packaging plasmid (psPAX2) and the envelope plasmid (pMD2.G). After lentiviral particle infection of JAWSII cells for 48 h, 15 µg/ml puromycin was utilized to select the positive clones, which were subsequently identified by RT-qPCR.

### Mouse models

There were five groups in each mouse model: no vaccine (No Vax), antigen plus alum (alum), antigen plus alum/CpG (alum/CpG), antigen plus alum/DP7 (alum/DP7) and antigen plus alum/CpG/DP7 (alum/CpG/DP7). To prepare the vaccines, 20 μg CpG was mixed with 40 μg DP7 at 37 °C for 15 min and then combined with 125 μg alum at 37 °C for 10 min, followed by incubation with 5 μg NY-ESO-1 protein or 10 μg OVA in PBS (pH = 7.4) in a total volume of 100 μl at room temperature for 10 min. Components were omitted at the appropriate steps for the single- and two-component formulations.

In the prophylactic tumor experiments, C57BL/6 mice were subcutaneously (s.c.) immunized three times with the above-described vaccines on days 0, 14, and 28. One week after the third immunization, C57BL/6 mice were challenged s.c. with 2 × 10^5^ NY-ESO-1-B16 cells or 2 × 10^6^ EG7-OVA cells in the back. The tumor volume was measured and calculated using the formula *V* = 0.5 × length (mm) × [width (mm)]^2^.

For the therapeutic tumor experiments, C57BL/6 mice were challenged s.c. with 2 × 10^5^ NY-ESO-1-B16 cells or 2 × 10^6^ EG7-OVA cells in the back on day 0. BALB/c mice were challenged s.c. with 2 × 10^5^ NY-ESO-1-4T1 cells in the back on day 0. Once the tumor volume was greater than 50 mm^3^, the tumor-bearing mice were randomly divided into five groups and then immunized with the above-described vaccines three times on days 5, 12, and 19. The tumor volumes were measured and calculated as described above.

### Statistical analysis

All statistical analyses were performed using GraphPad Prism 6.0 (GraphPad Software, La Jolla, USA). Differences among two groups were analyzed using one-way ANOVA unless otherwise indicated. Values of *P* < 0.05 were considered statistically significant.

### Reporting summary

Further information on research design is available in the Nature Research Reporting Summary linked to this article.

## Supplementary information

Supplementary Information

Reporting Summary

## Data Availability

The datasets generated and/or analyzed during the current study are available from the corresponding author upon reasonable request
