# Supplementary material for: Rational design of innate defense regulator peptides as tumor vaccine adjuvants
Source: NPJ Vaccines. 2021 May 20;6:75. doi: 10.1038/s41541-021-00334-3 (PMC8138013; doi:10.1038/s41541-021-00334-3)
Supplement: Supplementary file 1 — Supplementary Information [file 41541_2021_334_MOESM1_ESM.pdf]

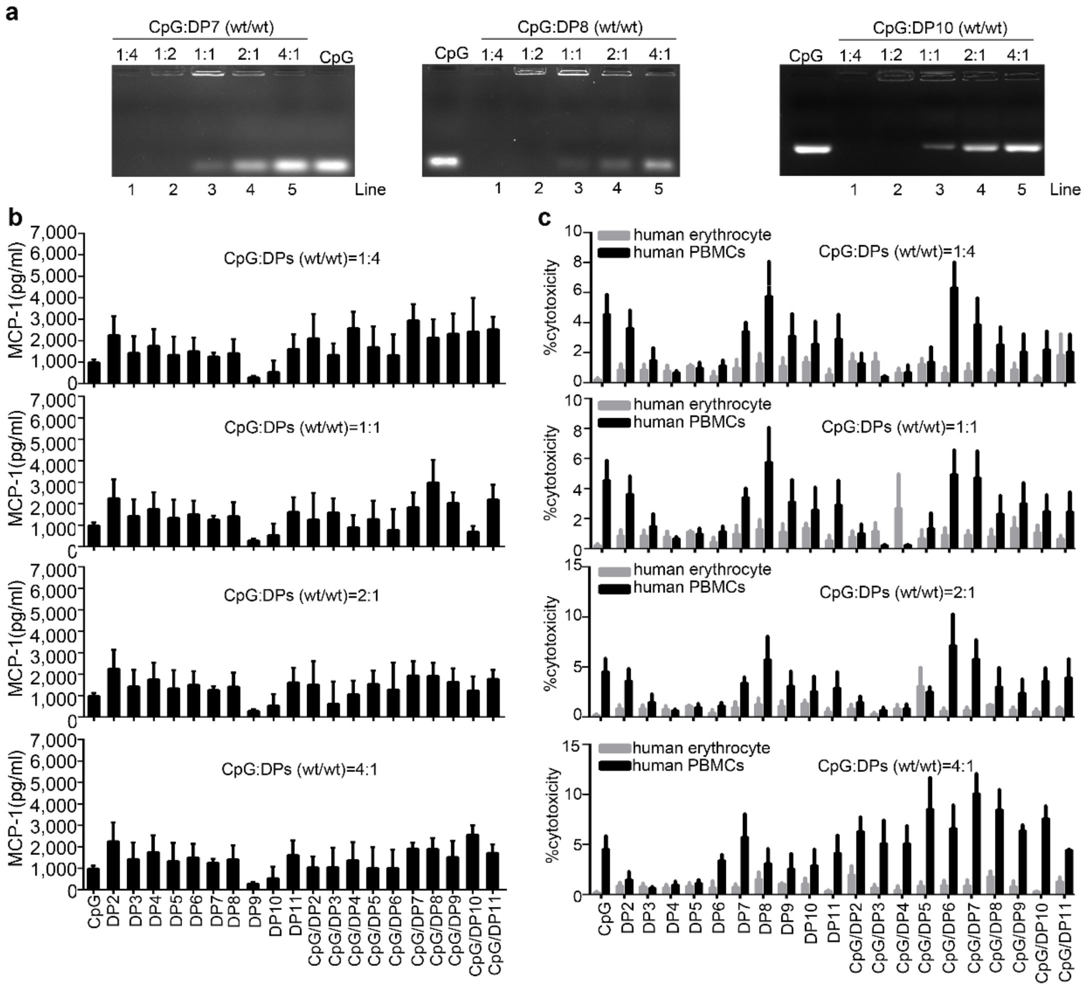

Supplementary Figure 1. *In vitro* preliminary screening of defense peptide candidates. **a** The representative image of AEMSA of CpG and DP7/DP8/DP10 complexes. CpG and DP7/DP8/DP10 were pre-complexed at various concentrations in PBS. Line 1:CpG(5μg)+ DP7/DP8/DP10 (20μg)；Line 2： CpG(5μg)+ DP7/DP8/DP10 (10μg)； Line 3: CpG(5μg)+ DP7/DP8/DP10 (5μg)； Line 4： CpG(5μg)+ DP7/DP8/DP10 (2.5μg)；Line 5: CpG(5μg) + DP7/DP8/DP10 (1.25μg). **b** PBMCs were stimulated with other ratios of CpG/DP2-11 (1:4,1:1,2:1,4:1; wt/wt) formulations for 24 h. The secretion of MCP-1 was detected. **c** PBMCs or human erythrocyte were incubated with other ratios of CpG/DP2-11 (1:4,1:1,2:1,4:1; wt/wt) for 24 hours. Then the LDH release from PBMCs and the total haemoglobin release from red blood cells were measured. N = 3 per group. Bars represent means and SEM.

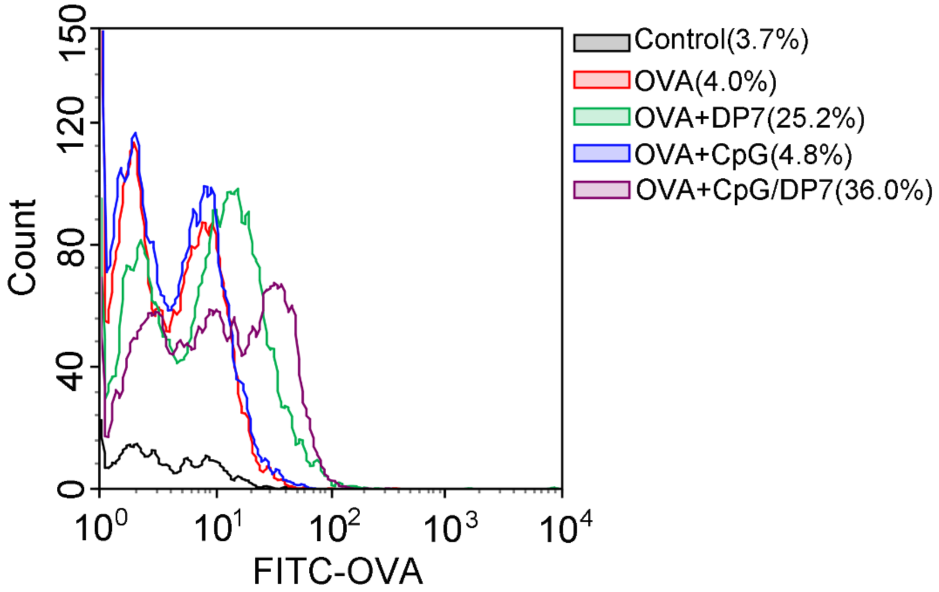

Supplementary Figure 2. CpG/DP7 complex enhanced the OVA uptake by BMDCs. BMDCs were stimulated with FITC-OVA in combination with CpG, DP7 or CpG/DP7 complex for 4 h. The cells were stained with CD11C-APC and analyzed by flow cytometry.

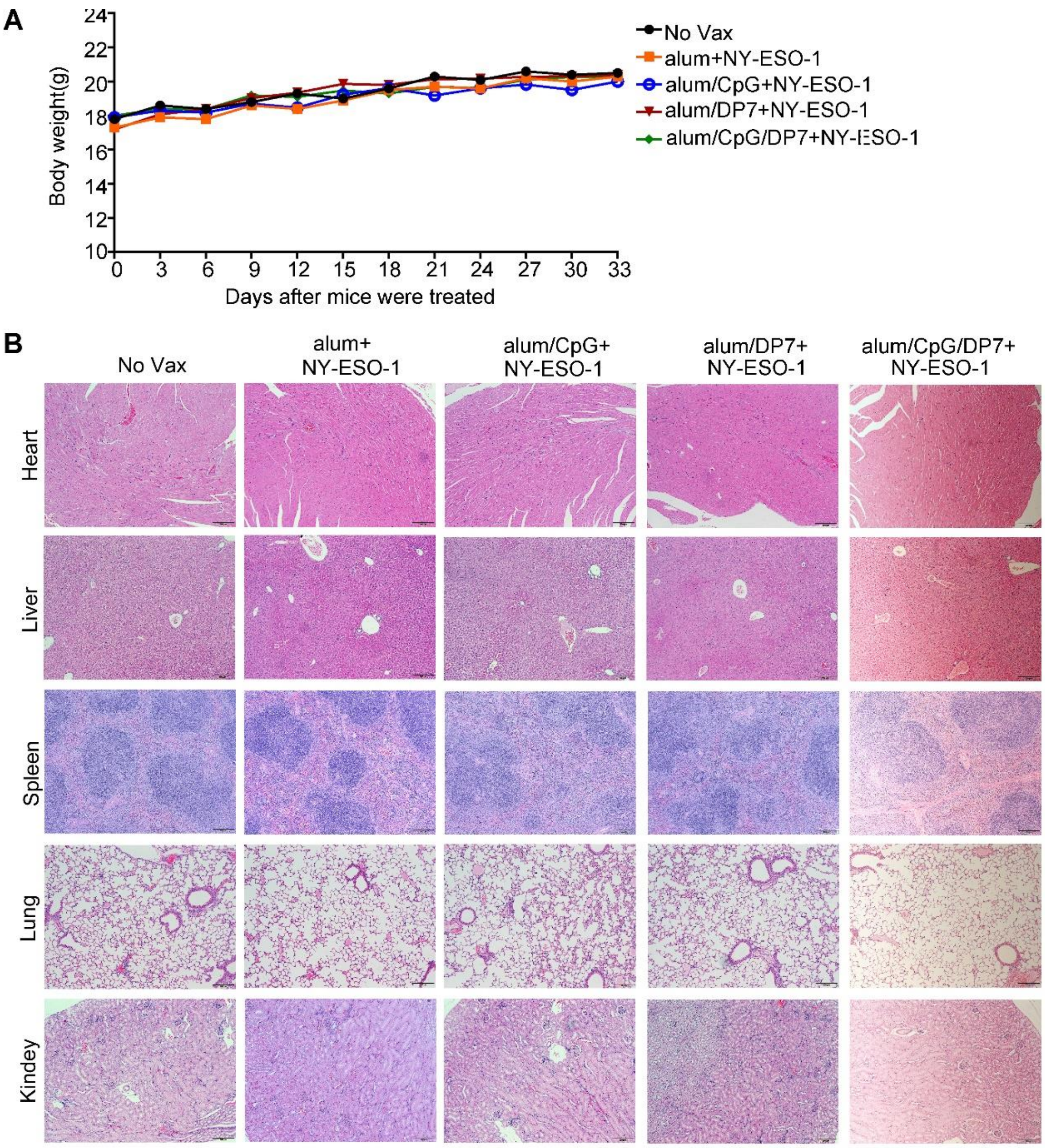

Supplementary Figure 3. Toxicity observation of NY-ESO-1 combined with the indicated adjuvants. Mice were immunized with NY-ESO-1 plus various adjuvants on day 0, 14 and 28. **a** The body weight of mice was monitored once every 3 days. **b** One week after the third immunization, the toxic and side effect on normal tissues including Heart, liver, spleen, lung and kidney were detected by histological analysis.

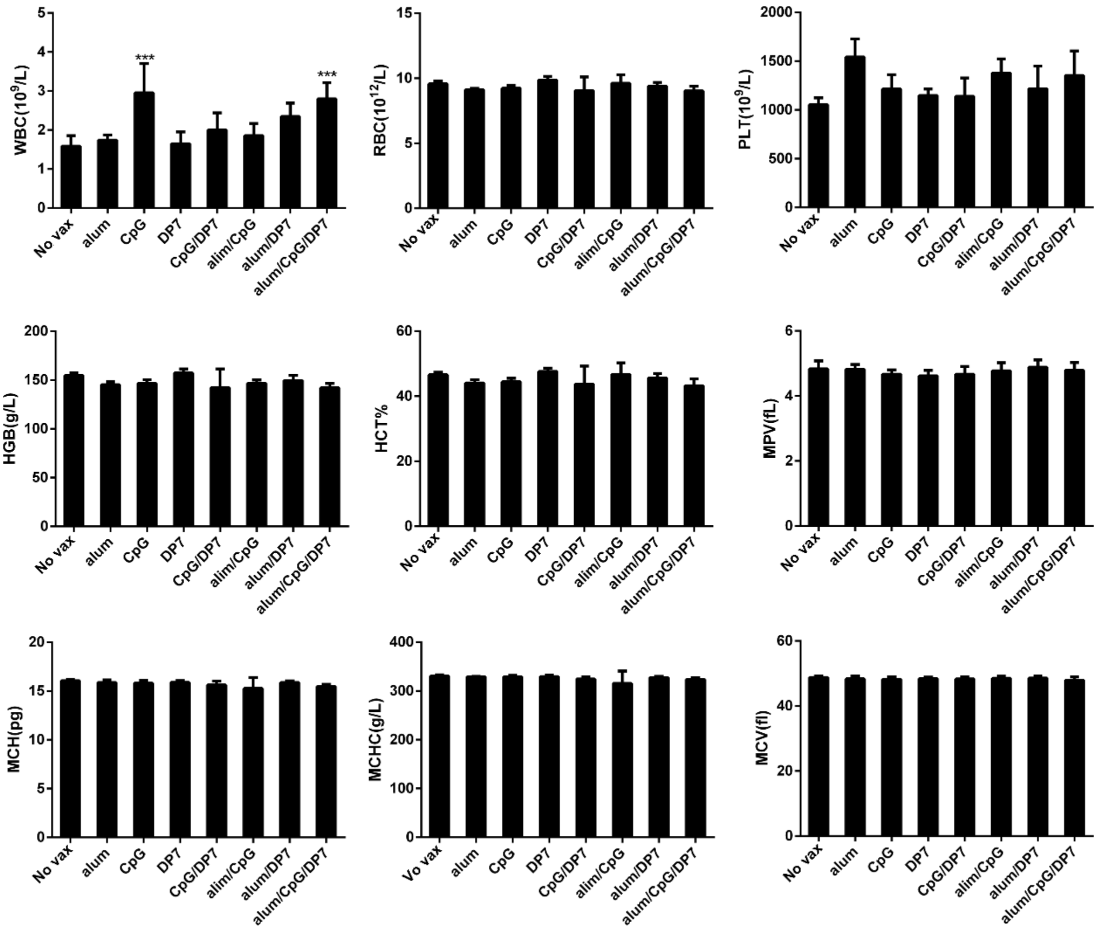

Supplementary Figure 4. Hematological studies of the indicated treatments and PBS. Mice were immunized with NY-ESO-1 plus the indicated adjuvants on day 0, 14 and 28. One week after the third immunization, hematology markers were monitored. Abbreviations: white blood cell count, WBC; red blood cell count, RBC; platelet, PLT; hemoglobin, HGB; hematocrit, HCT; mean platelet volume, MPV; mean corpuscular hemoglobin, MCH; mean corpuscular hemoglobin concentration, MCHC; mean corpuscular volume, MCV; N = 5~6 per group. Bars represent means and SEM. *P*<0.001, ***.

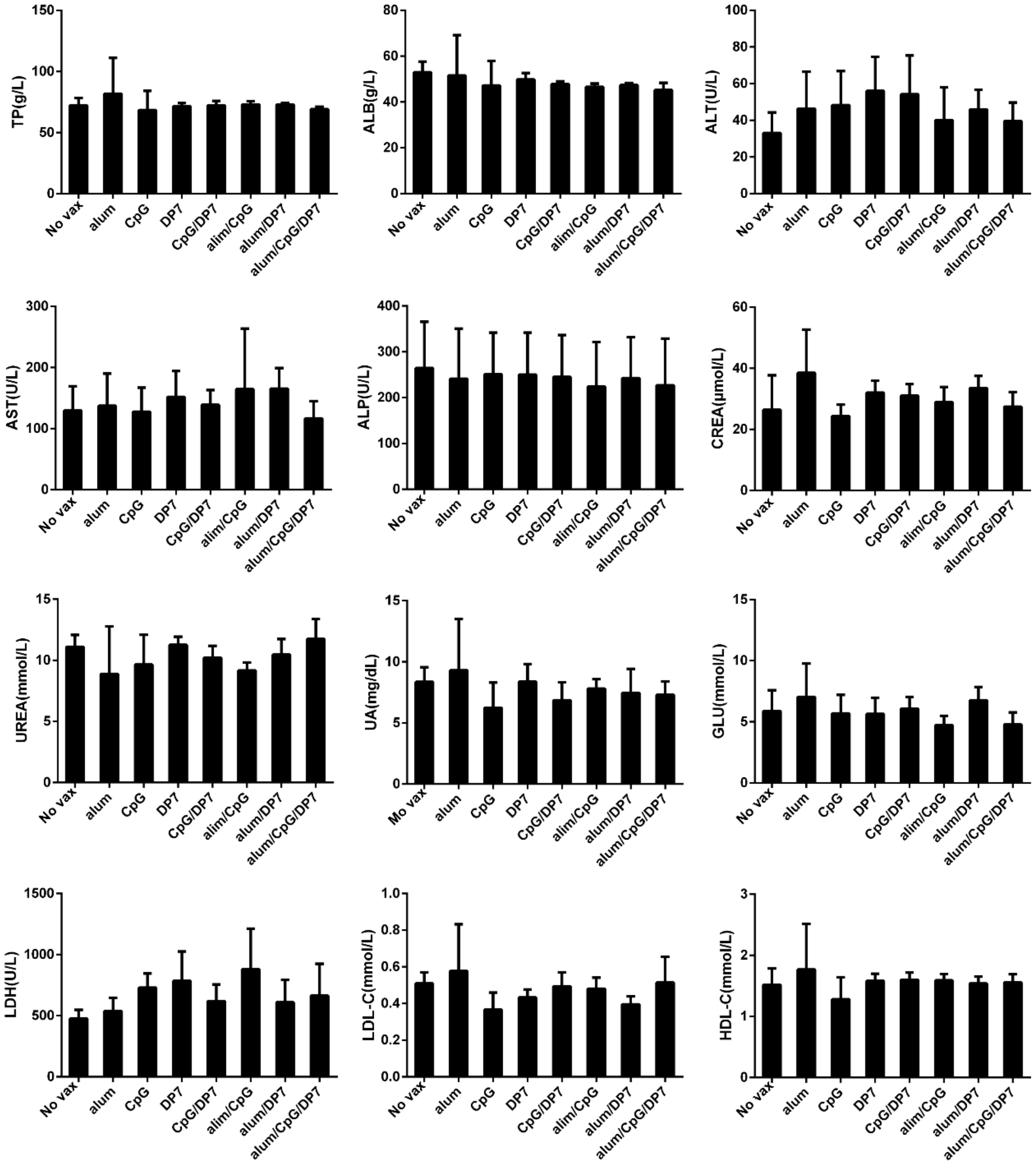

Supplementary Figure 5. The studies of serum biochemical biomarkers. Mice were immunized with NY-ESO-1 plus the indicated adjuvants on day 0, 14 and 28. One week after the third immunization, the serum biochemical biomarkers were measured. Abbreviations: total protein, TP; albumin, ALB; alanine aminotransferase, ALT; aspartate transaminase, AST; alkaline phosphatase, ALP; creatinine, CREA; urea, UREA; uric acid, UA; glucose, GLU; lactate dehydrogenase, LDH; high-density lipoprotein cholesterol, HDL-C; low-density lipoprotein cholesterol, LDL-C. N = 5~6 per group. Bars represent means and SEM.

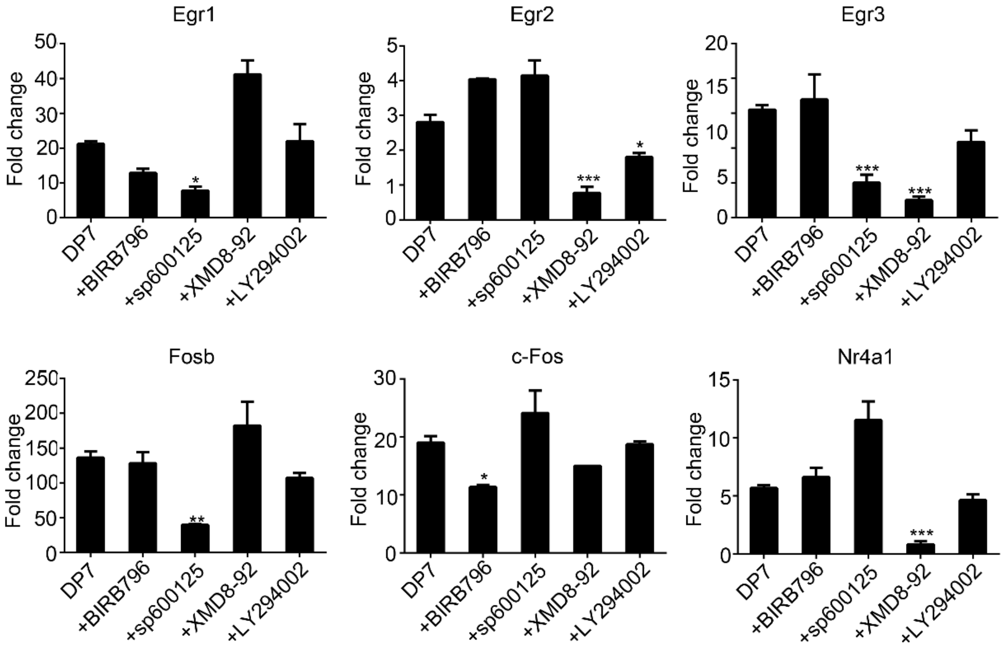

Supplementary Figure 6. Signaling pathways which were not involved in DP7 activity. JAWSII cells were pre-treated by chemical inhibitors, stimulated by DP7(40 μg/ml) for 30 min and then harvested for RT-PCR analysis. *P*<0.05, *; *P*<0.01, **; *P*<0.001, ***. Data are representative of three experiments with three replicates each. Bars represent means and SEM.

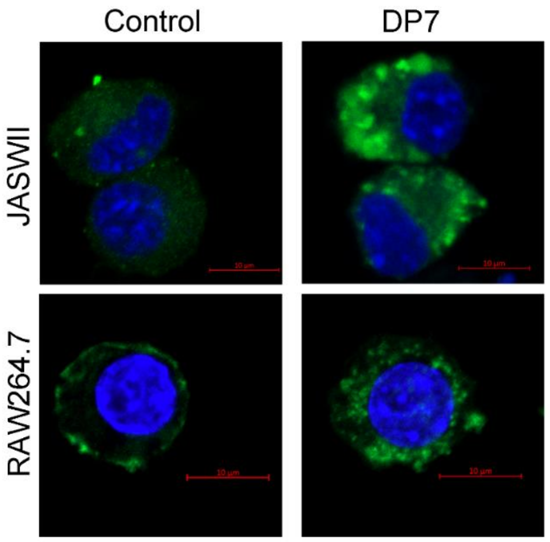

Supplementary Figure 7. Representative fluorescence images of GPR35 internalization in JASWII and RAW264.7 cells. JASWII and RAW264.7 cells were treated with DP7 for 30 min. After fixed, cells were stained with anti-GPR35 and then

secondary antibodies labeled with FITC(Green). The nucleus was stained with DAPI (blue).

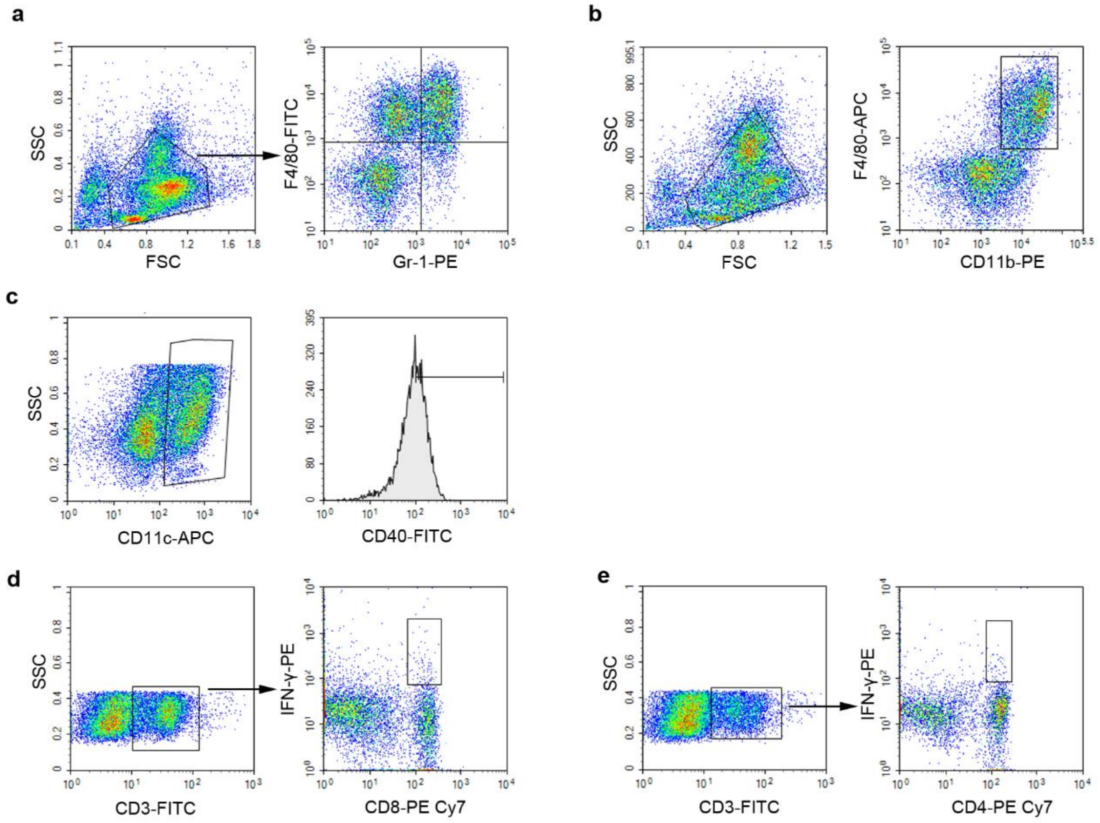

Supplementary Figure 8. Gating strategy by flow cytometry. a and **b** For leukocyte recruitment in Fig.1d, cells were first gated by SSC-FSC then Gr1, F4/80 or CD11b. Neutrophils, inflammatory monocytes and macrophages were gated as $Gr1^{+}F4/80^{-}$, $F4/80^{+}Gr1^{+}$ and $F4/80^{+}CD11b^{+}$ cells, respectively. **c** For BMDC maturation in Fig.2b, BMDCs were first gated by SSC-FSC. BMDCs were gated as $CD11c^{+}$ and analyzed for CD40 expression. The analysis for CD80 and CD86 expression is same as above. **d** and **e** For IFN-γ intracellular staining in Fig.4c and d, cells were first gated by SSC-FSC and then T cells in mouse splenocytes were gated as $CD3^{+}$. $CD4^{+}$ IFN-γ^{+} or $CD8^{+}$ IFN-γ^{+} were gated from $CD3^{+}$ cells to analyze the IFN-γ expression.

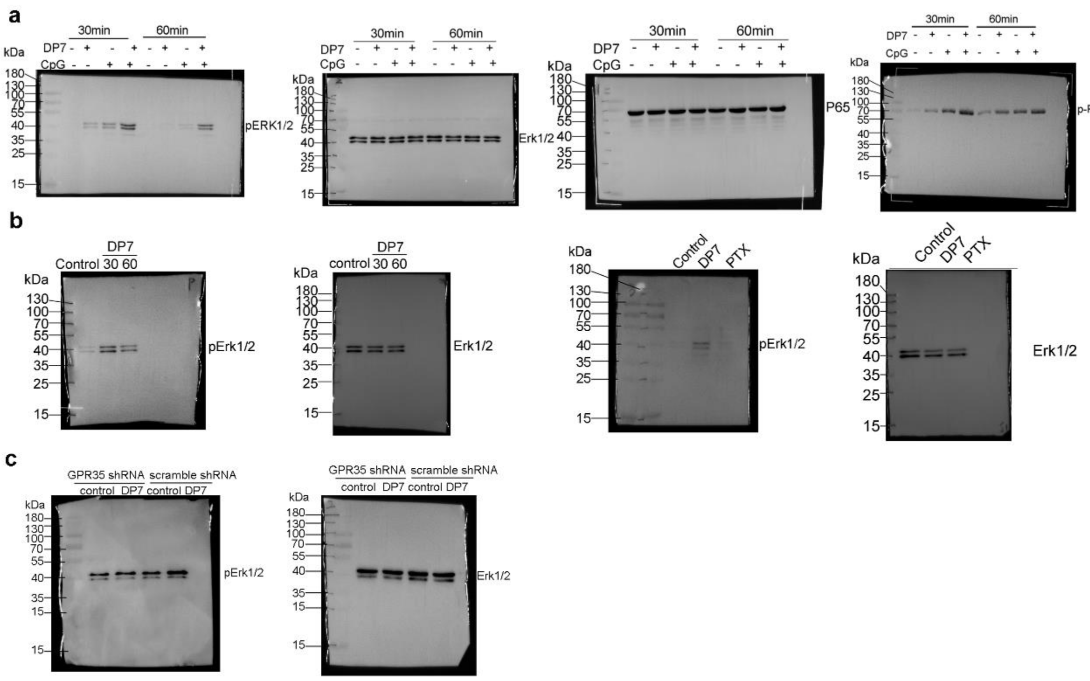

Supplementary Figure 9. Full, uncropped blot from Fig.2e(**a**), Fig.6b(**b**) and Fig.7c(**c**)with molecular weight marker.

Supplementary Table 1: The information of defense peptides

Peptides	Sequences	MW	Theoretical pI
Bac2A	RLARIVVIRVAR	1421.77	12.88
DP2	VQWRIRVCVIRA	1498.84	12.1
DP3	VQWRIRIAVIRA	1480.80	12.8
DP4	VCWRIRVAVIRA	1441.78	12.1
DP5	VQLRIRVCVIRR	1510.89	12.4
DP6	KQWRIRVAVIRA	1495.81	12.7
DP7	VQWRIRVAVIRK	1523.86	12.7
DP8	VQLRIRVCVIRK	1482.88	12.11
DP9	KQWRIRVCVIRA	1527.88	12.11
DP10	VQLRCRVCVIRK	1472.86	11.24
DP11	VQWRIRIAVIRK	1537.89	12.7

Supplementary Table 2. The list of up-regulated genes by DP7 in the RNA-seq analysis

Gene ID	Gene name	Descrption	Fold change
14282	Fosb	protein fosB	114.79
15370	Nr4a1	hormone receptor|nuclear hormone receptor NUR/77	19.81
13653	Egr1	Krox-24 nuclear protein|early growth response protein 1	16.63
13655	Egr3	early growth response protein 3	15.7
14281	Fos	cellular oncogene fos	6.34
236904	Klhl15	-	3.73
21872	Tjp1	tight junction protein ZO-1	3.44
620246	Gpr52	probable G-protein coupled receptor 52	3.32
15936	Ier2	CHX1|T-lymphocyte-activated protein	3.15
14857	Gsta1	GST class-alpha member	3.08
66071	Ethe1	ethylmalonic encephalopathy protein 1 homologmitochondrial	2.89
381809	Clec4b2	antigen presenting cell lectin-like receptor A1	2.82
109594	Lmo1	LIM domain only protein 1	2.81
12795	Plk3	FGF-inducible kinasekinase PLK3	2.75
211770	Trib1	trib1|tribbles homolog 1	2.69
622976	Gm6377	uncharacterized protein LOC622976	2.68
16069	Igj	J chain|immunoglobulin J chain	2.68
12227	Btg2	B-cell translocation gene 2TG2	2.47
18413	Osm	oncostatin-M	2.42
380921	Dgkh	diacylglycerol kinase eta	2.4
13654	Egr2	E3 SUMO-protein ligase EGR2	2.4
67287	Parp6	ADP-ribosyltransferase diphtheria toxin-like 17	2.3
50754	Fbxw7	F-box and WD-40 domain protein 7Fbxw6	2.25
14066	F3	tissue factor	2.23
19132	Prph	peripherin 1	2.22
64095	Gpr35	G-protein coupled receptor 35	2.2
226551	Suco	SUN domain-containing ossification factor	2.19
67144	Lrrc40	leucine-rich repeat-containing protein 40	2.19
320595	Phf8	histone lysine demethylase PHF8	2.17

268706	Slc38a9	putative sodium-coupled neutral amino acid transporter 9	2.15
1E+08	Gm12359	-	2.13
218630	Ccno	UDG2|cyclin U|cyclin-O|cyclin-like uracil-DNA glycosylase	2.1
108829	Jmjd1c	jumonji domain-containing protein 1C	2.09
100986	Akap9	A-kinase anchor protein 9	2.08
67200	Ccdc77	coiled-coil domain-containing protein 77	2.06
228413	Prrg4	proline-rich Gla protein 4	2.05
64058	Perp	keratinocyte-associated protein 1associated target	2.05
214359	Tmem51	-	2.05
52335	Atxn1l	ataxin-1-like	2.04
668415	Gm9159	-	2.03
212285	Arap2	PARX protein	2.02
18227	Nr4a2	NUR-related factor 1	2.02
21930	Tnfaip6	TNF alpha-induced protein 6	2.01
230676	Szt2	Seizure threshold 2 protein	2.01
277854	Depdc5	DEP domain-containing protein 5	2.01
