# Supplementary material for: Rational design of innate defense regulator peptides as tumor vaccine adjuvants
Source: NPJ Vaccines. 2021 May 20;6:75. doi: 10.1038/s41541-021-00334-3 (PMC8138013; doi:10.1038/s41541-021-00334-3)
Supplement: Supplementary file 2 — Reporting Summary [file 41541_2021_334_MOESM2_ESM.pdf]

nature research

Corresponding author(s): Li Yang; NPJVACCINES-00816R

Last updated by author(s): 2021-4-1

Reporting Summary

Nature Research wishes to improve the reproducibility of the work that we publish. This form provides structure for consistency and transparency in reporting. For further information on Nature Research policies, see our Editorial Policies and the Editorial Policy Checklist.

Statistics

For all statistical analyses, confirm that the following items are present in the figure legend, table legend, main text, or Methods section.

n/a	Confirmed	
☐	☒	The exact sample size (n) for each experimental group/condition, given as a discrete number and unit of measurement
☐	☒	A statement on whether measurements were taken from distinct samples or whether the same sample was measured repeatedly
☐	☒	The statistical test(s) used AND whether they are one- or two-sided<br>*Only common tests should be described solely by name; describe more complex techniques in the Methods section.*
☐	☒	A description of all covariates tested
☒	☐	A description of any assumptions or corrections, such as tests of normality and adjustment for multiple comparisons
☐	☒	A full description of the statistical parameters including central tendency (e.g. means) or other basic estimates (e.g. regression coefficient) AND variation (e.g. standard deviation) or associated estimates of uncertainty (e.g. confidence intervals)
☒	☐	For null hypothesis testing, the test statistic (e.g. F, t, r) with confidence intervals, effect sizes, degrees of freedom and P value noted<br>*Give P values as exact values whenever suitable.*
☒	☐	For Bayesian analysis, information on the choice of priors and Markov chain Monte Carlo settings
☒	☐	For hierarchical and complex designs, identification of the appropriate level for tests and full reporting of outcomes
☒	☐	Estimates of effect sizes (e.g. Cohen's d, Pearson's r), indicating how they were calculated

Our web collection on statistics for biologists contains articles on many of the points above.

Software and code

Policy information about availability of computer code

Data collection	All laboratory data were collected in Manuscript.
Data analysis	Statistical analyses were performed using GraphPad Prism 6.0.

For manuscripts utilizing custom algorithms or software that are central to the research but not yet described in published literature, software must be made available to editors and reviewers. We strongly encourage code deposition in a community repository (e.g. GitHub). See the Nature Research guidelines for submitting code & software for further information.

Data

Policy information about availability of data

All manuscripts must include a data availability statement. This statement should provide the following information, where applicable:

- Accession codes, unique identifiers, or web links for publicly available datasets
- A list of figures that have associated raw data
- A description of any restrictions on data availability

The datasets generated and/or analyzed during the current study are available from the corresponding author upon reasonable request.

Field-specific reporting

Please select the one below that is the best fit for your research. If you are not sure, read the appropriate sections before making your selection.

☒ Life sciences ☐ Behavioural & social sciences ☐ Ecological, evolutionary & environmental sciences

For a reference copy of the document with all sections, see nature.com/documents/nr-reporting-summary-flat.pdf

Life sciences study design

All studies must disclose on these points even when the disclosure is negative.

Sample size	No sample-size calculation was performed. For in vivo studies, each group used N=10 mice, the sample size was chosen based on previous papers (Li, M., et al. Mol Cancer 13, 179 (2014)). The number of tumor cells that were subcutaneously (s.c.) injected to mice was determined based on previous papers (Li, M., et al. Mol Cancer 13, 179 (2014)) and the results of pretesting.
Data exclusions	No data were excluded.
Replication	Samples from Chemokine and cytokine release, Cytotoxicity assay, Leukocyte recruitment, Measurement of antibody titers, IFN-γ intracellular staining and ELISpot assay reported as mean of 3 technical replicates.Samples from Quantitative real-time PCR were generally analysed in a single experimental run, and reported as three experiments with three replicates each.
Randomization	Animals were randomized into the study groups at time of immunization.
Blinding	Blinding was not used in our experiments. Staff involved in animal study ,data generation and analysis were not blinded as only objective quantitative measurements were performed.

Reporting for specific materials, systems and methods

We require information from authors about some types of materials, experimental systems and methods used in many studies. Here, indicate whether each material, system or method listed is relevant to your study. If you are not sure if a list item applies to your research, read the appropriate section before selecting a response.

Materials & experimental systems

n/a	Involved in the study	
☐	☒	Antibodies
☐	☒	Eukaryotic cell lines
☒	☐	Palaeontology and archaeology
☐	☒	Animals and other organisms
☒	☐	Human research participants
☒	☐	Clinical data
☒	☐	Dual use research of concern

Methods

n/a	Involved in the study	
☒	☐	ChIP-seq
☐	☒	Flow cytometry
☒	☐	MRI-based neuroimaging

Antibodies

Antibodies used

1: goat anti-GPR35 (clone M-14; Santa Cruz Biotechnology Cat# sc-79507; 1:100)
2: FITC-anti-mouse CD3ε(clone 145-2C11; BD Biosciences Cat# 553062)
3: PE-Cy7-anti-mouse CD8α (clone 53-6.7; BD Biosciences Cat# 552877)
4: PE-Cy7-anti-mouse CD4(clone RM4-5; BD Biosciences Cat# 552775)
5: PE-anti-mouse IFN-γ(clone XMG1.2; BD Biosciences Cat# 554412)
6: Goat Anti-Mouse IgG, Human ads-HRP (SourhernBiotech Cat#1030-05)
7: Goat Anti-Mouse IgG1, Human ads-HRP (SourhernBiotech Cat#1070-05)
8: Goat Anti-Mouse IgG2c, Human ads-HRP (SourhernBiotech Cat#1079-05)
9: NF-κB p65 XP® Rabbit mAb (clone D14E12; Cell Signaling Technology Cat#8242)
10: Phospho-NF-κB p65 (Ser536) Rabbit mAb (clone 93H1; Cell Signaling Technology Cat#3033)
11: Phospho- Erk1/2 (Thr202/Tyr204) XP® Rabbit mAb(clone D13.14.4E ; Cell Signaling Technology Cat#4370)
12: Erk1/2 Rabbit mAb (clone 137F5; Cell Signaling Technology Cat#4695)
13: FITC Rat Anti-Mouse CD40 (Clone 3/23; BD Biosciences Cat#561845)
14: PerCP-Cy5.5 Hamster Anti-Mouse CD80(clone 16-10A1; BD Biosciences Cat# 560526)
15: FITC Rat Anti-Mouse CD86 (clone GL1; BD Biosciences Cat# 561962)
16: PE Rat Anti-Mouse Gr-1(clone RB6-8C5; BD Biosciences Cat# 553128)
17: APC Rat Anti-Mouse F4/80(clone T45-2342; BD Biosciences Cat# 566787)
18: PE Rat Anti-CD11b (clone M1/70; BD Biosciences Cat# 557397)
19: APC-anti-Mouse CD11c(Clone HL3; BD Biosciences Cat# 550261)

Validation

1: goat anti-GPR35 (clone M-14; Santa Cruz Biotechnology Cat# sc-79507; 1:100)
Reactivity: Mouse and Rat; Application: Western Blotting, immunofluorescence and solid phase ELISA.
2: FITC-anti-mouse CD3ε(clone 145-2C11; BD Biosciences Cat# 553062)
Reactivity: Mouse; Application: Flow cytometry$Fluorescence microscopy.
3: PE-Cy7-anti-mouse CD8α (clone 53-6.7; BD Biosciences Cat# 552877)
Reactivity Mouse ;Application Flow cytometry
4: PE-Cy7-anti-mouse CD4(clone RM4-5; BD Biosciences Cat# 552775)
Reactivity Mouse ;Application Flow cytometry
5: PE-anti-mouse IFN-γ(clone XMG1.2; BD Biosciences Cat# 554412)
Reactivity Mouse ; Application Intracellular staining (flow cytometry)
6: Goat Anti-Mouse IgG, Human ads-HRP (SourhernBiotech Cat#1030-05)
Specificity: Reacts with the heavy chains of mouse IgG1, IgG2a, IgG2b, IgG2c, and IgG3
Applications: Quality tested applications for relevant formats include -ELISA, FLISA,Flow Cytometry,Other referenced applications for relevant formats include -
ELISpot,Immunohistochemistry-Frozen Sections,Immunohistochemistry-Paraffin Sections, Immunocytochemistry, Western Blot, Multiplex
7: Goat Anti-Mouse IgG1, Human ads-HRP (SourhernBiotech Cat#1070-05)
Specificity: Reacts with the heavy chain of mouse IgG1
Applications: Quality tested applications for relevant formats include -ELISA, FLISA,Flow Cytometry,Other referenced applications for relevant formats include -
ELISpot,Immunohistochemistry-Frozen Sections,Immunohistochemistry-Paraffin Sections,Immunohistochemistry-Whole Mount ,Immunocytochemistry,Electron Microscopy ,Western Blot,Surface Plasmon Resonance , Multiplex
8: Goat Anti-Mouse IgG2c, Human ads-HRP (SourhernBiotech Cat#1079-05)
Specificity: Reacts with the heavy chain of C57BL/6 mouse IgG2c
Applications: Quality tested applications for relevant formats include -ELISA, FLISA, Other referenced applications for relevant formats include -
ELISpot, Flow Cytometry, Immunohistochemistry-Paraffin Sections, Western Blot, Depletion.
9: NF-κB p65 XP® Rabbit mAb (clone D14E12; Cell Signaling Technology Cat#8242)
Application Key:Western, Immunoprecipitation , Immunohistochemistry,Chromatin Immunoprecipitation ,Immunofluorescence, Flow Cytometry
Species Cross-Reactivity Key: Human, Mouse, Rat, Hamster, Monkey, Dog
10: Phospho-NF-κB p65 (Ser536) Rabbit mAb (clone 93H1; Cell Signaling Technology Cat#3033)
Application Key: Western, Immunoprecipitation, Immunofluorescence, Flow Cytometry
Species Cross-Reactivity Key: Human, Mouse, Rat, Hamster, Monkey, Pig
11: Phospho- Erk1/2 (Thr202/Tyr204) XP® Rabbit mAb(clone D13.14.4E ; Cell Signaling Technology Cat#4370)
Application Key: Western, Immunoprecipitation, Immunohistochemistry, Immunofluorescence, Flow Cytometry
Species Cross-Reactivity Key: Human, Mouse, Rat, Hamster, Mink, D.melanogaster, Monkey, Dog, Zebrafish, Bovine, Pig ,S. cerevisiae
12: Erk1/2 Rabbit mAb (clone 137F5; Cell Signaling Technology Cat#4695)
Application Key: Western, Immunoprecipitation, Immunohistochemistry, Immunofluorescence, Flow Cytometry
Species Cross-Reactivity Key: Human, Mouse, Rat, Hamster, Mink, D.melanogaster, Monkey, Dog, Zebrafish, Bovine, Pig , C. elegans
13: FITC Rat Anti-Mouse CD40 (Clone 3/23; BD Biosciences Cat#561845)
Reactivity: Mouse; Application: Flow cytometry.
14: PerCP-Cy5.5 Hamster Anti-Mouse CD80(clone 16-10A1; BD Biosciences Cat# 560526)
Reactivity: Mouse; Application: Flow cytometry.
15: FITC Rat Anti-Mouse CD86 (clone GL1; BD Biosciences Cat# 561962)
Reactivity: Mouse; Application: Flow cytometry.
16: PE Rat Anti-Mouse Gr-1(clone RB6-8C5; BD Biosciences Cat# 553128)
Reactivity: Mouse; Application: Flow cytometry.
17: APC Rat Anti-Mouse F4/80(clone T45-2342; BD Biosciences Cat# 566787)
Reactivity: Mouse; Application: Flow cytometry.
18: PE Rat Anti-CD11b (clone M1/70; BD Biosciences Cat# 557397)
Reactivity: Mouse Human; Application: Flow cytometry.
19: APC-anti-Mouse CD11c(Clone HL3; BD Biosciences Cat# 550261)
Reactivity: Mouse; Application: Flow cytometry.

Eukaryotic cell lines

Policy information about cell lines

Cell line source(s)

B16-F10 melanoma cells, 4T1 breast cancer cells, OVA-transfected OVA-E.G7 T lymphoma cells and JAWSII cells were obtained from American Type Culture Collection (ATCC, Manassas, VA).

Authentication

No formal authentication was carried out. Cell morphology and adhesion was consistent with expectations.B16F10 and 4T1 expressing NY-ESO-1 was evaluated for uniform expression of NY-ESO-1 ;

Mycoplasma contamination

Cell lines were not tested for mycoplasma.

Commonly misidentified lines
(See ICLAC register)

Not applicable.

Animals and other organisms

Policy information about studies involving animals; ARRIVE guidelines recommended for reporting animal research

Laboratory animals	Female ,6- to 8-week-old ,C57BL/6 and BALB/c mice
Wild animals	not applicable
Field-collected samples	not applicable
Ethics oversight	All animal experiments and protocols used in this study were approved by the Ethics Review Committee for Animal Experimentation of Sichuan University

Note that full information on the approval of the study protocol must also be provided in the manuscript.

Flow Cytometry

Plots

Confirm that:

☒ The axis labels state the marker and fluorochrome used (e.g. CD4-FITC).

☒ The axis scales are clearly visible. Include numbers along axes only for bottom left plot of group (a 'group' is an analysis of identical markers).

☒ All plots are contour plots with outliers or pseudocolor plots.

☒ A numerical value for number of cells or percentage (with statistics) is provided.

Methodology

Sample preparation	BMDCs were isolated from C57BL/6 mice and cultured in the presence of 10 ng/ml recombinant murine GM-CSF and IL-4 .BMDCs from 5-day-old cultures were collected afor further experiments. After sacrifice, spleens are removed from the mice, without fat tissue attached. Mouse splenocytes were isolated by lymphocytes separating solution.
Instrument	BD FACSCalibur
Software	BD FACSCalibur software was used to acquire data. Further data analysis was performed using NovoExpress software.
Cell population abundance	Not applicable as cell sorting was not performed
Gating strategy	For leukocyte recruitment :<br>cells were first gated by SSC-FSC then Gr1, F4/80 or CD11b. Neutrophils, inflammatory monocytes and macrophages were gated as Gr1+F4/80-, F4/80+Gr1+ and F4/80+CD11b+ cells, respectively.<br>For BMDC maturation , BMDCs were first gated by SSC-FSC. BMDCs were gated as CD11c+ and analyzed for CD40 expression. The analysis for CD80 and CD86 expression is same as above.<br>For IFN-γ intracellular staining , cells were first gated by SSC-FSC and then T cells in mouse splenocytes were gated as CD3 +. CD4+ IFN-γ+ or CD8+ IFN-γ+ were gated from CD3ε+ cells to analyze the IFN-γ expression. Strategy is exemplified in Supplementary Figure 8.

☒ Tick this box to confirm that a figure exemplifying the gating strategy is provided in the Supplementary Information.
